# Single‐Crystalline Borate Covalent Organic Frameworks for Solid‐State Lithium Metal Batteries

**DOI:** 10.1002/advs.202513879

**Published:** 2026-01-20

**Authors:** Ye Tian, Xiaolong Cheng, Lei Cheng, Yide Chang, Jixin Wu, Muhua Gu, Ki‐Taek Bang, Rui Wang, Ran Tao, Yufeng Wang, Soonyong So, Yanming Wang, Yoonseob Kim

**Affiliations:** ^1^ Department of Chemical and Biological Engineering The Hong Kong University of Science and Technology Kowloon Hong Kong SAR P. R. China; ^2^ Global Institute of Future Technology Shanghai Jiao Tong University Shanghai P. R. China; ^3^ Department of Chemistry The University of Hong Kong Hong Kong SAR P. R. China; ^4^ Hydrogen Energy Research Center Korea Research Institute of Chemical Technology Daejeon South Korea; ^5^ Energy Institute The Hong Kong University of Science and Technology Hong Kong SAR P. R. China

**Keywords:** ionic covalent organic frameworks, lithium metal batteries, lithium‐ion conduction, quasi‐solid‐state electrolyte

## Abstract

To advance lithium metal batteries, novel solid‐state electrolytes are crucial. Covalent Organic Frameworks (COFs) are promising due to their crystalline, porous structure, light composition, strong bonds, high surface area, and stability. COFs can be engineered for enhanced ion conduction, with their porosity and ion‐functional groups enabling fast ion movement and uniform lithium deposition. We synthesized a single‐crystalline 3D borate COF (B‐COF), achieving an ionic conductivity of 8.1 mS cm^−1^ at room temperature and a lithium‐ion transference number of 0.98 in a quasi‐solid‐state. In symmetric cells, B‐COF supported stable lithium deposition/stripping for 2000 h, suppressing dendrite formation. Full cells with LiFePO_4_ cathodes cycled stably at 0.5C, delivering 147 mAh g^−1^ initial capacity, 91.8% retention, and 99.98% Coulombic efficiency over 600 cycles. These results highlight B‐COF's potential as a high‐performance solid electrolyte for lithium metal batteries.

## Graphical abstract

Advancing lithium metal batteries demands novel solid‐state electrolytes. Covalent organic frameworks (COFs) offer high ion conduction via porosity and functional groups. We synthesized single‐crystalline 3D borate COF with 8.1 mS cm^−1^ conductivity and 0.98 Li^+^ transference. It enables dendrite‐free cycling for 2000 h and stable LiFePO_4_ cells over 600 cycles with 91.8% retention.

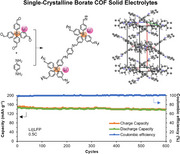

The development of lithium metal batteries (LMBs) is vital for high‐energy‐density applications like electric vehicles and large‐scale energy storage. However, LMBs face challenges, including lithium dendrite formation, which poses safety risks, and unstable lithium‐electrolyte interfaces, causing rapid degradation [1, 2, 3]. Solid‐state electrolytes that transport Li^+^ rapidly and selectively at ambient temperature are a solution. Materials like polymers, ceramics, metal–organic frameworks, and covalent organic frameworks (COFs) have been developed [4, 5, 6, 7]. Each has pros and cons, but here we focus on developing ion‐conductive COFs (iCOFs) as novel electrolytes. COFs are crystalline, porous materials with lightweight composition, strong covalent bonds, high surface area, stability, and low density [6, 8, 9, 10]. Their designable structure and ion‐conductive moieties make iCOFs effective electrolytes, achieving high conductivity (*σ*) and single‐ion transference number (*t*_Li+_) [11, 12, 13, 14].

Since 2015, Zhang's group pioneered borate COFs for Li^+^ conduction, sparking interest; the COF‐based solid electrolyte research developed rapidly, as 2D COFs offered high Li^+^ conductivity via *π*–*π* stacked, 1D ion channels [15]. For example, Hu et al. reported an imidazolium iCOF with *σ* of 7.2 mS cm^−1^ and *t*_Li+_ of 0.81 at room temperature (r.t.) [16]. Yuan et al. reported that ether chain functionalized imidazolate COFs to show a high *σ* of 8.81 mS cm^−1^ and a transference number of 0.974 [17]. Adding polyethylene glycol chains to COFs enhances ionic bond dissociation, improving solid‐state battery performance [18]. However, most of those COFs are defect‐containing and polycrystalline; thus, their structures are not fully understood, and their full properties are not realized (Figure 1a) [19, 20, 21]. Compared to the 2D COFs, 3D COFs tend to form higher crystallinity, including single crystals, which can significantly reduce resistances arising from intraparticle intergrain disorders (Figure 1b) [22, 23, 24]. Moreover, the ordered structures of iCOFs contribute to the uniform deposition of Li^+^, leading to dendrite suppression and sustained battery life [25].

**FIGURE 1:**
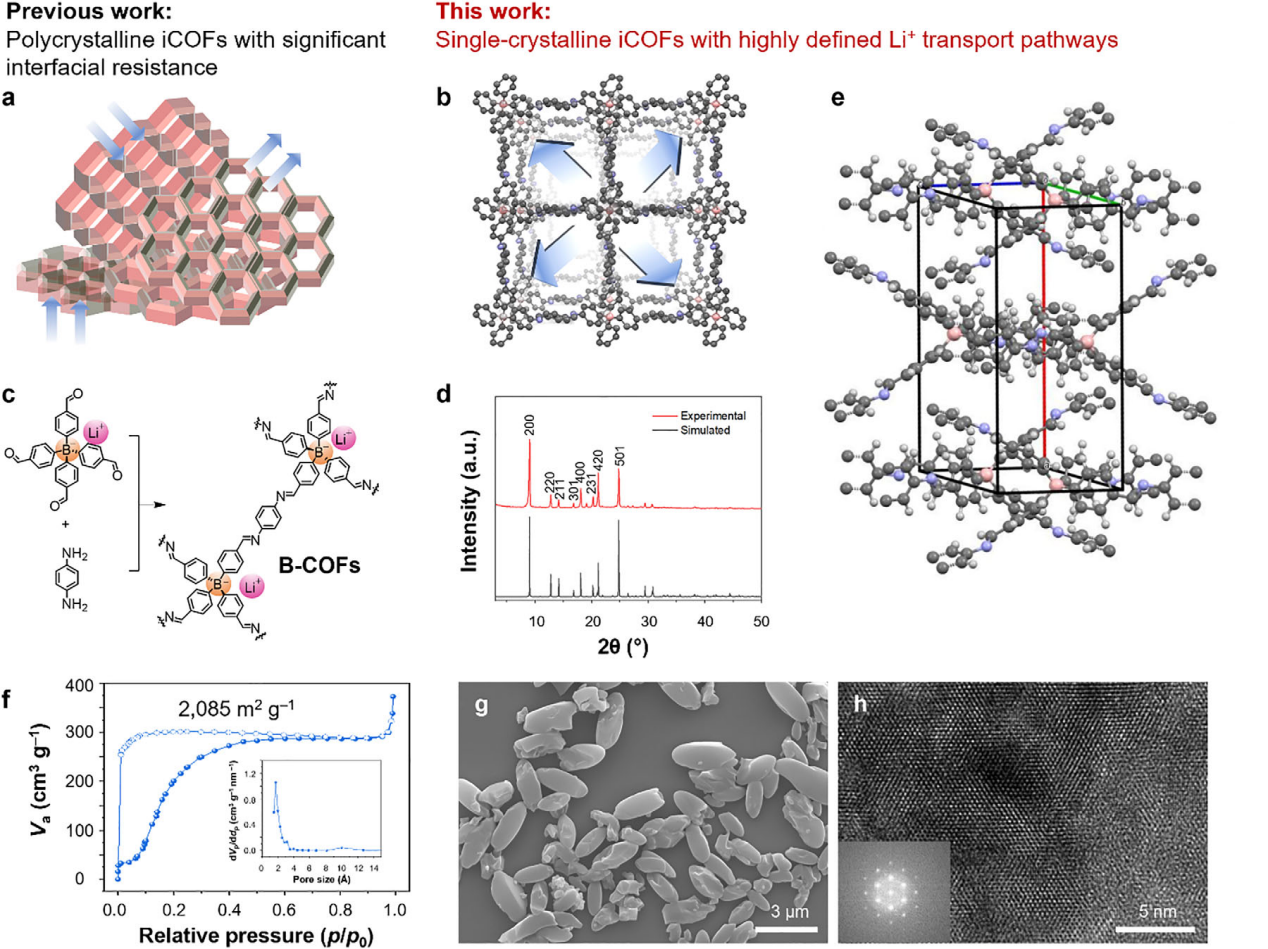
Schematics of crystalline 3D borate covalent organic frameworks (B‐COFs) and their structural characterization. (a) Schematic of Li^+^ transport in previously studied 2D polycrystalline iCOFs. (b) Schematic of Li^+^ transport in 3D single crystalline B‐COFs, C, gray; N, blue; B, orange. (c) Schiff base condensation reaction scheme between lithium tetrakis(4‐formylphenyl)borate and *p*‐phenylenediamine to yield B‐COFs. (d) Experimental powder X‐ray diffraction (PXRD) data of B‐COF. (e) Single crystal cell structure of B‐COF obtained by Micro Electron Diffraction. Gray, blue, light pink, and white, for C, N, B, and H, respectively. (f) Nitrogen adsorption and desorption isotherms of B‐COF. Inset shows the pore size distribution. (g) SEM image of the B‐COF crystals. (h) High‐resolution transmission electron microscopy image of B‐COF. Inset shows the corresponding fast Fourier transform patterns.

Thus, we synthesized 3D iCOF (Figure 1), utilizing the ***dia*** topological used in COF‐303 as a template [26], while using ionic tetrahedral building blocks to construct iCOFs. Specifically, tetrahedral lithium tetrakis(4‐formylphenyl)borate monomers and *p*‐phenylenediamine monomers were reacted under solvothermal conditions to result in the B‐COFs in microcrystal forms with ca. 2 µm length (Figure 1; Schemes S1–S3 and Figures S1–S3; See Supporting Information for synthetic details). The chemical bond structure of B‐COF was confirmed by Fourier Transform Infrared Spectroscopy (FT‐IR; Figure S4). B‐COFs exhibited a single crystalline feature as reported in the COF‐303. The diffraction peaks from the B‐COFs were very sharp and nearly identical to those of COF‐303, indicating they had the same single crystallinity (Figure 1d). Solid‐state ^11^B and ^7^Li nuclear magnetic resonance revealed boron and lithium species at −9.65 ppm, and −0.28, respectively. (Figure S5). The crystal structure of the B‐COF sample was successfully elucidated through Micro electron diffraction, showing that the B‐COF crystallizes in the tetragonal system, specifically in the I41/a space group, and the symmetry operations of the B‐COF crystal structure include a fourfold screw axis and an a‐glide plane (Figure 1e; Figures S6–S10 and Tables S1–S6). The permanent porosity of synthesized B‐COF was analyzed through N_2_ adsorption measurements at 77 K. The N_2_ adsorption isotherm revealed the microporous structure of B‐COFs, showing typical hysteresis of COF‐303 due to the dynamic response of the framework (higher gas pressures activate the pores), and the calculated Brunauer–Emmett–Teller surface area was 2085 m^2^ g^−1^, and the average pore diameter was 2.5 nm (Figure 1f).

Electron microscopy revealed the morphology of B‐COFs. Scanning electron microscopy (SEM) showed uniformly sized rice grain‐like single crystals around 2 µm synthesized without modulators (Figure 1g). Although we can enlarge the crystal size (with reduced homogeneity), we focused on working with those small and uniform‐sized crystals for better packing into electrolyte applications (See Figure S22 and Supporting Information for more discussion). The highly ordered periodic structure of B‐COF was also evident in high‐resolution transmission electron microscopy (HR‐TEM) images and electron diffraction patterns (Figure 1h; Figures S3 and S6). Lattice fringes were observed along the crystals, with spacings of 3.05 nm (Figure S6a,b). The uniform and symmetric electron diffraction patterns indicated the single‐crystalline nature of the 3D B‐COF crystals. Large‐scale optical microscopy images also showed highly uniform 2 µm B‐COF microcrystals over a large scale (Figure S6c). Additionally, SEM EDX, TEM energy dispersive X‐ray spectroscopy (EDX), and inductively coupled plasma optical emission spectrometry (ICP‐OES) characterizations support the single‐crystalline nature of the B‐COFs (Figures S12 and S13 and Table S7). Even those B‐COF crystals are chemically stable in various solvents, such as acetone, methanol, 2 m HCl aqueous solution, 2 m NaOH aqueous solution (Figure S14), and thermally stable up to 418°C (Figure S15).

Subsequently, we evaluated the electrochemical performance of B‐COF as a solid‐state electrolyte. B‐COF crystals were cold‐pressed into electrolyte pellets and tested in coin cells using stainless steel as electrodes. The pure B‐COF pellet showed a *σ* of 0.004 mS cm^−1^ at r.t. (Figure S16). This value did not meet the minimum *σ* requirements for LMB operation. Thus, we added 30 wt.% of LiTFSI salts to enhance ion transport properties (hereafter, we used this sample for testing unless otherwise noted and named as Li^+^@B‐COF). With LiTFSI salt, *σ* increased to 0.59 mS cm^−1^ with 30 wt.% addition, then decreased to 0.4 mS cm^−1^ with 50 wt.% (Figures S16a and S19). Adding 50 wt.% LiTFSI made an excess amount of TFSI^−^ in the electrolytes, resulting in a lower *t*_Li+_ of 0.8. Electrochemical impedance spectroscopy (EIS) of B‐COF displayed Nyquist behavior (Figure S16b). An Arrhenius plot was created to show an extremely low activation energy of only 0.07 eV (Figures S16c and S17). Another critical parameter for assessing the conductive behavior of the electrolyte is the average *t*_Li+_ value. A high *t*_Li+_ value, typically over 0.8, indicates *σ* primarily arising from Li^+^; values over 0.9 signify single‐ion transport behavior. The *t*_Li+_ of Li^+^@B‐COF, determined using the Bruce–Vincent–Evans technique in a Li metal symmetric cell, was 0.98 (Figure 2a).

**FIGURE 2:**
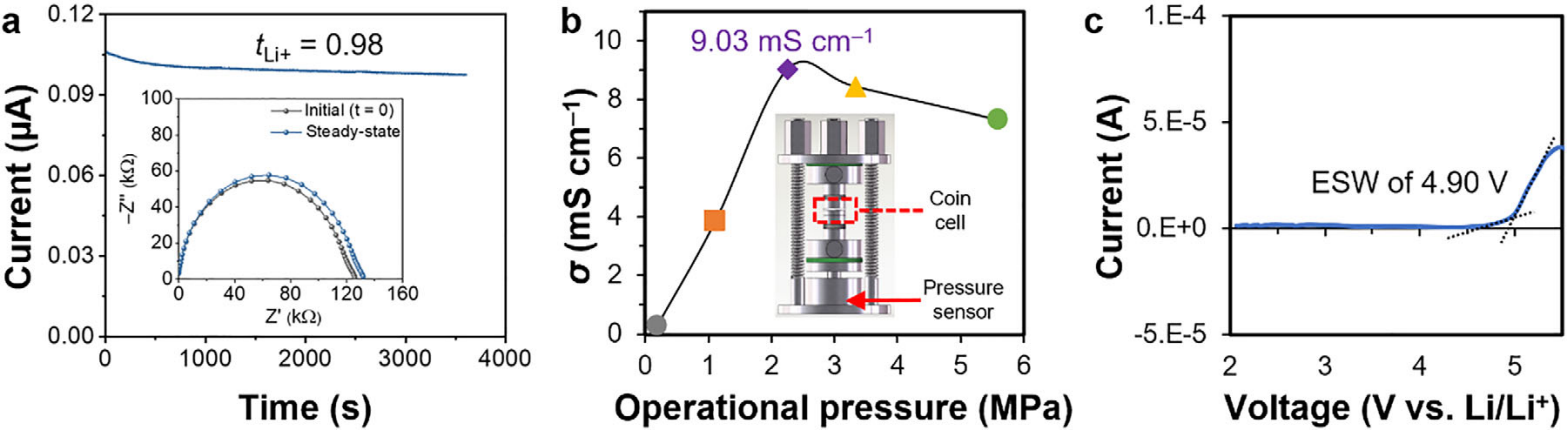
Electrochemical performance of B‐COFs. (a) Current‐time plots and calculated *t*_Li+_ of B‐COF‐based symmetric cells. (b) Ionic conductivities of pure B‐COF under pressures. (c) LSV test for Li^+^@B‐COF electrolyte.

To further enhance the Li^+^ conduction of B‐COF electrolytes in battery cells, we added 5 wt.% plasticizer polycarbonate (PC) at both interfaces of the B‐COF electrolyte with the electrode, totaling 10 wt.% in a cell. This 10 wt.% is a minimal amount of liquid when making quasi‐solid‐state electrolytes, excluding the possibility of Li^+^ conduction in the liquid phase [27]. Other quasi‐solid‐state battery cells typically have 20 wt.% of organic solvents [17]. After adding the plasticizer as an interface modifier, the *σ* at r.t. of Li^+^@B‐COF electrolyte significantly increased to 8.1 mS cm^−1^. (Figure S16d) Compared to other reported iCOF‐based electrolytes, our Li^+^@B‐COF exhibited exceptionally high *σ* and *t*_Li+_, with less Li salt and additives (Table S8). Considering the particles' crystalline nature, we further experimented with external pressure to reduce the interparticle resistance of B‐COF electrolytes. The pellet from pure B‐COFs, without salts and PC, was pressed, and as the pressure increased, the *σ* gradually increased and peaked at 9.03 mS cm^−1^ at 2.26 MPa (Figure 2b; Figure S18). Typically, ceramics have greater than 100 MPa external operating pressure [28]. External pressure of 2.26 MPa is very low and practically applicable to the cells. Linear Sweep Voltammetry (LSV), measured from 1.0 to 5.5 V vs. Li^+^/Li (scan rate: 1 mV s^−1^) with a stainless‐steel|pellet|Li cell at r.t., indicated that Li^+^@B‐COF has a stable oxidation stability window up to 4.90 V (Figure 2c).

Given the efficient conduction of Li^+^@B‐COF electrolytes, we utilized them as quasi‐solid‐state electrolytes to assess their performance in battery cells without external pressure. We assembled symmetric coin cells to characterize the stability of the Li^+^@B‐COF electrolyte interface with Li metal. Constant current charge/discharge tests at a current density of 0.1 mA cm^−2^ were applied to the symmetric cells. During a test period of up to 2000 h, the symmetric cells exhibited stable lithium plating and stripping behavior with a small overpotential of 100 mV (Figure 3a). Then, we assembled LMB full cells with LiFePO_4_ as the cathode (Li|Li^+^@B‐COF|LFP). Battery's rate performance at 2.5–4.0 V was 136, 128, 116, and 90 mAh g^−1^ at 0.2, 0.5, 1, and 2 C, respectively (Figure 3b; Figure S20). Cycling test at 0.5 C showed an initial discharge capacity of 147 mAh g^−1^, then only 8.2% decreased after 600 cycles, with a Coulombic efficiency remaining at 99.98% (Figure 3c,d). The stable B‐COFs were paired with the NCM811 cathode to show stable operation up to 100 cycles when tested at 0.1 C: initial capacity was 165 mAh g^−1^ and >96.4% remained (Figure 3e).

**FIGURE 3:**
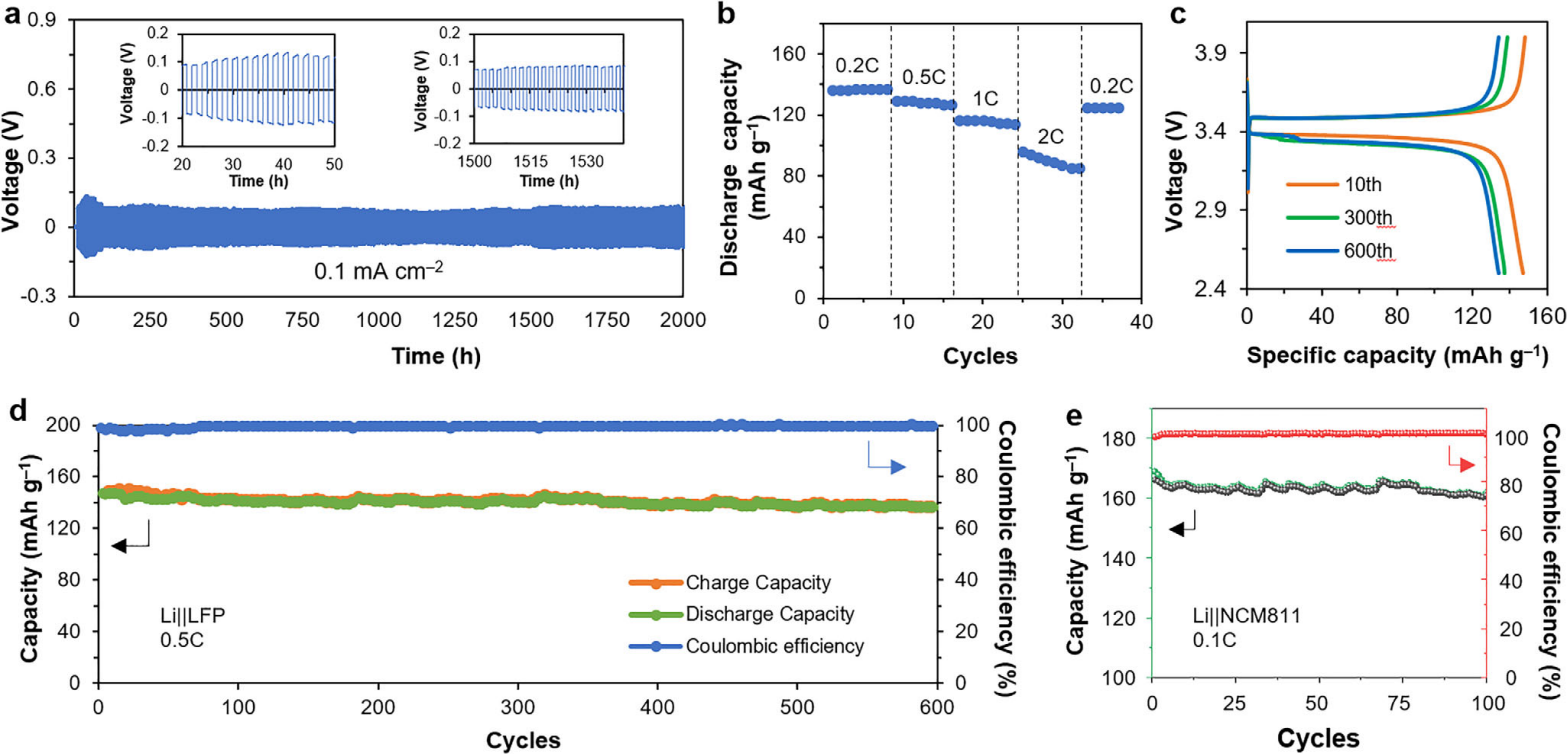
Lithium metal batteries using the Li^+^@B‐COF. (a) Galvanostatic cycling at a current density of 0.1 mA cm^−2^ in Li||Li symmetric cells with Li**^+^** @B‐COF. (b) Rate performance of Li||LFP full‐cell charged to 4.0 V with Li^+^@B‐COF at increasing rates from 0.2 C to 2 C, then back to 0.2 C. (c) Li||LFP full cell charge and discharge curves at selected cycles with Li^+^@B‐COF at 0.5 C. (d) Cycling in Li||LFP cells charged to 4.0 V with Li^+^@B‐COF at 0.5 C. (e) Cycling in Li||NCM811 cells charged to 4.3 V with Li^+^@B‐COF at 0.5 C.

Post‐mortem studies revealed that the Li metal electrode interface remained flat with no significant dendrite growth after extended Li^+^ stripping and deposition cycles, even at a high current density of up to 1 mA cm^−2^ (Figures S21–S23 and S25 and S26). This is due to the fact that single‐crystalline 3D B‐COF provided continuous ion transport pathways, and uniformly sized particles showed minimal interparticle resistances, thus promoting uniform Li^+^ deposition and effectively suppressing dendrite growth. As shown in SEM imaging, we had very uniform particles, tightly packed in the electrolytes (Figure S22a,b). In contrast, a control with larger, non‐uniform crystals showed unstable charge–discharge curves and higher overpotentials (Figure S22c), with a lower initial discharge capacity of 60 mAh g^−1^ and only 130 cycles (Figure S22d–f). Using electron backscatter diffraction (EBSD) (Figure 4a,b; Figure S14), we analyzed the microcrystalline structure of the lithium metal. Inverse pole figure and contrast maps (Figure 4c–e) revealed epitaxial deposition at the Li|Li^+^@B‐COF interface along the (101) crystal plane. Small grains (∼15 µm) were observed beneath the lithium at the electrolyte‐electrode interface, confirming uniform Li^+^ deposition through the B‐COF electrolyte, resulting in homogeneous microstructural patterns.

**FIGURE 4:**
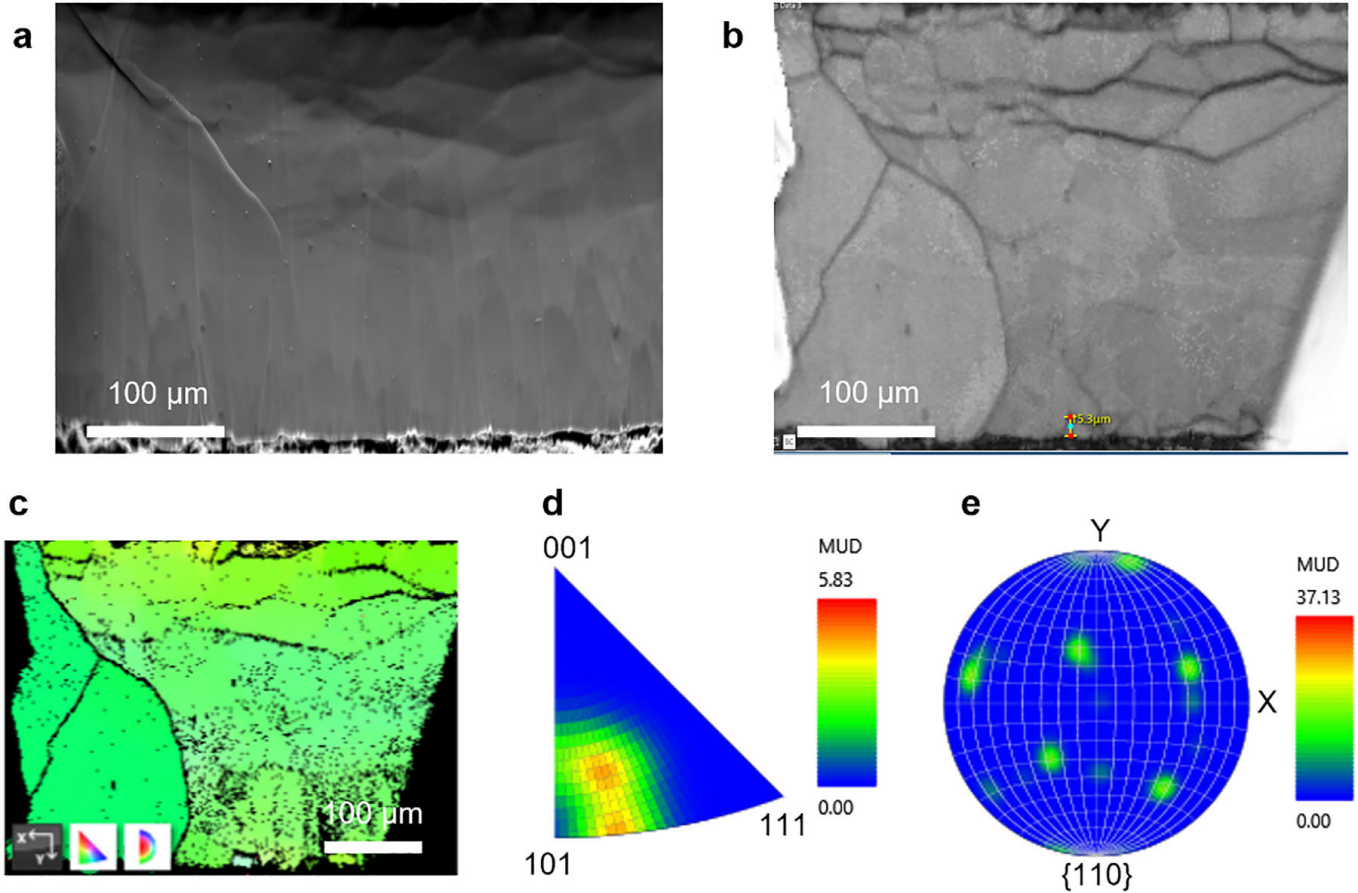
Analysis of the microstructural evolution during lithium electrodeposition. (a) SEM image of a fresh, polished cross‐section of a lithium sample. (b) Band contrast image of EBSD tested plated lithium at the Li|Li^+^@B‐COF interface. (c) EBSD inverse pole figure (IPF) map of plated lithium at the Li|Li^+^@B‐COF interface. (d,e) IPF pole figure, and the corresponding orientation distribution function.

We conducted computational studies on Li^+^ binding and transport in anionic B‐COF compared to neutral COF‐303 (Figure 5; Figures S27 and S28 and Tables S9 and S10). Calculations showed Li^+^ binding energy with B‐COF at −6.047 eV, stronger than that of COF‐303 and LiTFSI (−5.379 eV), enabling better dissociation of LiTFSI and enhanced Li^+^ mobility. This dissociation is key to superior ion‐transport performance in B‐COF. To address potential Li^+^ trapping at boron sites, density functional theory (DFT) and Climbing Image Nudged Elastic Band (CI‐NEB) methods revealed a two‐step hopping mechanism in B‐COF with barriers of 0.370–0.380 and 0.220–0.357 eV, similar to COF‐303's single‐step barrier (0.341–0.395 eV), ensuring high mobility. Electrostatic potential (ESP) analysis indicated negative regions around B centers in B‐COF (−120 kcal mol^−1^), stabilizing Li^+^ and guiding migration through 3D channels, reducing desolvation energy. This Li^+^ incorporation attenuates ESP gradients, boosting conductivity. Neutral COFs show uniform ESP (−20 to +20 kcal mol^−1^), resulting in higher barriers and lower efficiency.

**FIGURE 5:**
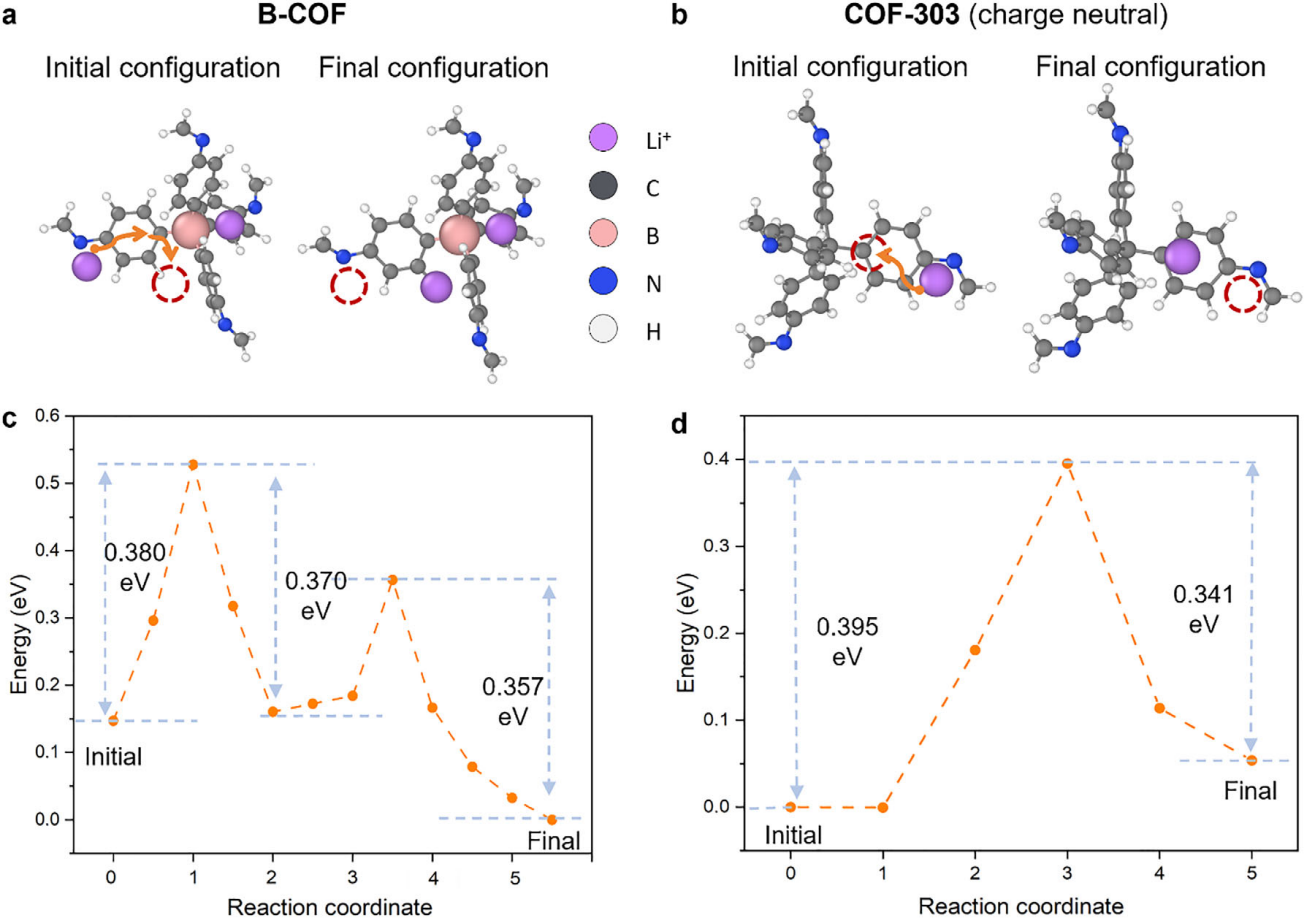
Model structures of B‐COF ((a) with one excess Li^+^), and COF‐303 ((b) charge‐neutral COFs with one excess Li^+^), and their calculated energy barriers along the reaction coordinates (c,d).

In this study, we successfully synthesized a single‐crystalline 3D B‐COF and demonstrated its exceptional potential as a high‐performance quasi‐solid‐state electrolyte for LMBs. The B‐COF exhibited remarkable ionic conductivity of 8.1 mS cm^−1^ at r.t. and an outstanding *t*_Li+_ number of 0.98, attributed to its highly ordered, porous structure and uniform ion‐conductive pathways. The single‐crystalline nature of the 3D B‐COF minimized interparticle resistance and facilitated uniform lithium deposition, effectively suppressing dendrite formation. Electrochemical tests in symmetric cells showed stable lithium plating/stripping for over 2000 h with low overpotential, while full cells with LiFePO_4_ cathodes delivered an initial capacity of 147 mAh g^−1^ at 0.5 C, retaining 91.8% capacity and 99.98% Coulombic efficiency after 600 cycles. Post‐mortem analyses confirmed the absence of significant dendrite growth, underscoring the B‐COF's ability to promote homogeneous lithium deposition. These findings highlight the transformative potential of single‐crystalline 3D B‐COFs as quasi‐solid‐state electrolytes, offering a promising pathway to address the safety and performance challenges of LMBs for high‐energy‐density applications such as electric vehicles and large‐scale energy storage.

## Author Contributions

Ye Tian synthesized the B‐COF materials and performed basic characterization of the materials and cells. Xiaolong Cheng helped make battery cells (symmetric and full), conducted related characterizations, and performed a part of the DFT calculations. Lei Cheng, Yide Chang, and Jixin Wu conducted thorough computational simulations. Muhua Gu helped with PXRD characterization and its simulation. Ki‐Taek Bang helped with the synthetic optimization of the B‐COFs. Rui Wang and Ran Tao helped with materials characterization. Yufeng Wang and Soonyong So helped with the characterization and understanding of the single crystallinity of the B‐COFs. Yanming Wang advised Lei, Yide, and Jixin on the computational work. Yoonseob Kim conceived and advised the project.

## Conflicts of Interest

The authors declare no conflicts of interest.

## Supporting information




**Supporting File**: advs73771‐sup‐0001‐SuppMat.docx.

## Data Availability

The data that support the findings of this study are available from the corresponding author upon reasonable request.
