# Supplementary material for: Single‐Crystalline Borate Covalent Organic Frameworks for Solid‐State Lithium Metal Batteries
Source: Adv Sci (Weinh). 2026 Jan 20;13(16):e13879. doi: 10.1002/advs.202513879 (PMC13042618; doi:10.1002/advs.202513879)
Supplement: Supplementary file 1 — Supporting File: advs73771‐sup‐0001‐SuppMat.docx. [file ADVS-13-e13879-s001.docx]

**Supporting Information**

**Single-Crystalline Borate Covalent Organic Frameworks for Solid-State Lithium Metal Batteries**

Ye Tian,^1,†^ Xiaolong Cheng,^1,†^ Lei Cheng,^2,†^ Yide Chang,^2^ Jixin Wu,^2^ Muhua Gu,^1^ Ki-Taek Bang,^1^ Rui Wang,^1^ Ran Tao,^1^ Yufeng Wang,^3^ Soonyong So,^4^ Yanming Wang,^2,^* and Yoonseob Kim^1,5,^*

^1^Department of Chemical and Biological Engineering, The Hong Kong University of Science and Technology, Clear Water Bay, Kowloon, Hong Kong SAR, 999077, China

^2^Global Institute of Future Technology, Shanghai Jiao Tong University, Shanghai 200240, People’s Republic of China

^3^Department of Chemistry, The University of Hong Kong, Hong Kong 999077, China

^4^Hydrogen Energy Research Center, Korea Research Institute of Chemical Technology, 141 Gajeongro, Yuseong, Daejeon 34114, South Korea

^5^Energy Institute, The Hong Kong University of Science and Technology, Hong Kong SAR, 999077, China

^†^These authors contributed equally to this work.

*To whom correspondence should be addressed: yanming.wang@sjtu.edu.cn, yoonseobkim@ust.hk

**Materials and Methods**

**Chemicals**

*p*-Phenylene diiodide (Aladdin, ACS reagent, ≥98%), *n*-butyl lithium (Sigma-Aldrich, ACS reagent, 2.0M in cyclohexane), boron chloride 1.0 M in heptane (Sigma-Aldrich, ACS reagent, 98%), *p*-phenylenediamine (Sigma-Aldrich, ACS reagent, 98%), mesitylene (Energy chemical, ACS reagent, 98%), 1,4-dioxane (Sigma-Aldrich, ACS reagent, 98%). Solvents used included acetone, tetrahydrofuran (THF), dimethyl sulfoxide (DMSO), dichloromethane (DCM), and dimethylformamide (DMF) were obtained from RCI Labscan Ltd. Ethanol was obtained from Honeywell. Deionized water (18.2 MΩ•cm) was used. All commercially available reagents and solvents were used as received without any further purification. Except THF and diethyl ether (Et_2_O) were dried with molecular sieves.

**General characterization method**

^1^H nuclear magnetic resonance (NMR) spectra were recorded on a Bruker AVII 400 MHz NMR Spectrometer. Chemical shifts are reported in delta (δ) units, expressed in parts per million (ppm) relative to the residual solvent as an internal standard (CDCl_3_, ^1^H: 7.26 ppm, ^13^C: 77.20 ppm; DMSO-*d_6_*, ^1^H: 2.50 ppm, ^13^C: 54.00 ppm; acetone-*d_6_*, ^1^H: 2.05 ppm, ^13^C: 206.70, 29.90 ppm). Field emission scanning electron microscopy (SEM) observations were performed on a JSM-7100F equipped with energy dispersive X-day (EDX) spectroscopy operated at an accelerating voltage of 15 kV. The samples were coated with gold before SEM and EDX measurement. Powder X-ray Diffraction (PXRD) data were collected on an analytical X-ray diffractometer (Cu Kα radiation λ = 1.54056 Å). Transmission electron microscopy images were obtained on a JEM-ARM200F (JEOL) operating at 200 kV, equipped with EDX spectroscopy operated at an accelerating voltage of 200 kV. The nitrogen adsorption-desorption isotherm was recorded on a MicrotacBEL-BelsorpminiX at 77K. Thermogravimetric analysis was measured with Discovery TGA5500 in the range of 25 to 900 °C with a heating rate of 10 °C min^–1^. The FTIR spectra were recorded by a Vertex 70 Hyperion 1000 (Bruker). The XPS was measured by a Kratos Axis Ultra DLD multi-technique surface analysis system.

**Single crystal analysis**

Electron diffraction data were collected by a JEM-2100 Plus transmission electron microscope manufactured by JEOL, operating under an accelerating voltage of 200 kV and a wavelength of 0.0025079 nm. The microscope is equipped with a MerelinEM high-speed direct electron camera. QUANTIFOIL copper grids (R1.2/1.3) and a Fischione 2550 cryo-transfer holder were utilized, with the environment maintained at a temperature of 77 K. A PELCO easiGlow™ glow discharge unit was employed for sample preparation.

The sample was prepared by dry-sprinkling the solid powder onto the copper grid. The procedure involved the following steps: 1. A suitable amount of the sample was placed in a centrifuge tube along with the copper grid and gently shaken to ensure the sample adhered to the grid. 2. The copper grid was then mounted onto the sample holder and inserted into the microscope's sample chamber. Liquid nitrogen was added to the dewar flask, allowing the temperature to decrease to 77 K. Once the desired temperature was achieved and the vacuum conditions stabilized, the measurements commenced.

Regarding the MicroED Data Collection and Analysis, the data were processed using XDS for diffraction data reduction. To enhance completeness, two sets of data (Table S5) were merged. The initial structure was solved using SHELXT, followed by refinement with SHELXL and OLEX 2. Relevant crystallographic and refinement details are summarized in Table S6.

**Synthesis of lithium tetrakis(4-iodophenyl)borate**

**Scheme S1. Synthetic procedure of lithium tetrakis(4-iodophenyl)borate.**

1,4-diiodo benzene (23.6 g; 100 mmol) was dissolved in dry Et_2_O (150 mL). The solution was cooled to –40 °C, and 2 M *n*-BuLi (50 mL; 100 mmol) was added dropwise while maintaining the temperature below −30 °C. The resulting slurry was stirred for 1 h at −40 °C and then cooled to −78 °C. Then, a 1 M solution of BCl_3_ in hexane (20 mL; 20 mmol) was added dropwise. A cooling bath was removed, and the mixture was allowed to warm to room temperature (r.t.) and left overnight. Solvents were evaporated under reduced pressure, and the solid residue was treated with a solution of aqueous LiCl (5 g in 50 mL of water). The crude product was filtered, washed with water (3 × 50 mL) and DCM (2 × 25 mL), and dried under reduced pressure. The dried product was dissolved in THF (100 mL). The resulting solution was filtered and evaporated. The pure anhydrous product was obtained by heating for 2 h at 150 °C under a high vacuum (10^−4^ Torr). White powder, dec. >355 °C, yield: 20.5 g (90%). ^1^H NMR (400 MHz, acetone-d_6_) δ 7.31 (dd, J = 8 Hz, 8H), 6.93–6.88 (m, 8H) ppm; MS (MALDI-TOF, m/z) calculated for C_24_ H_16_ BI_4_^–^, 820.74; found, 820.7671.


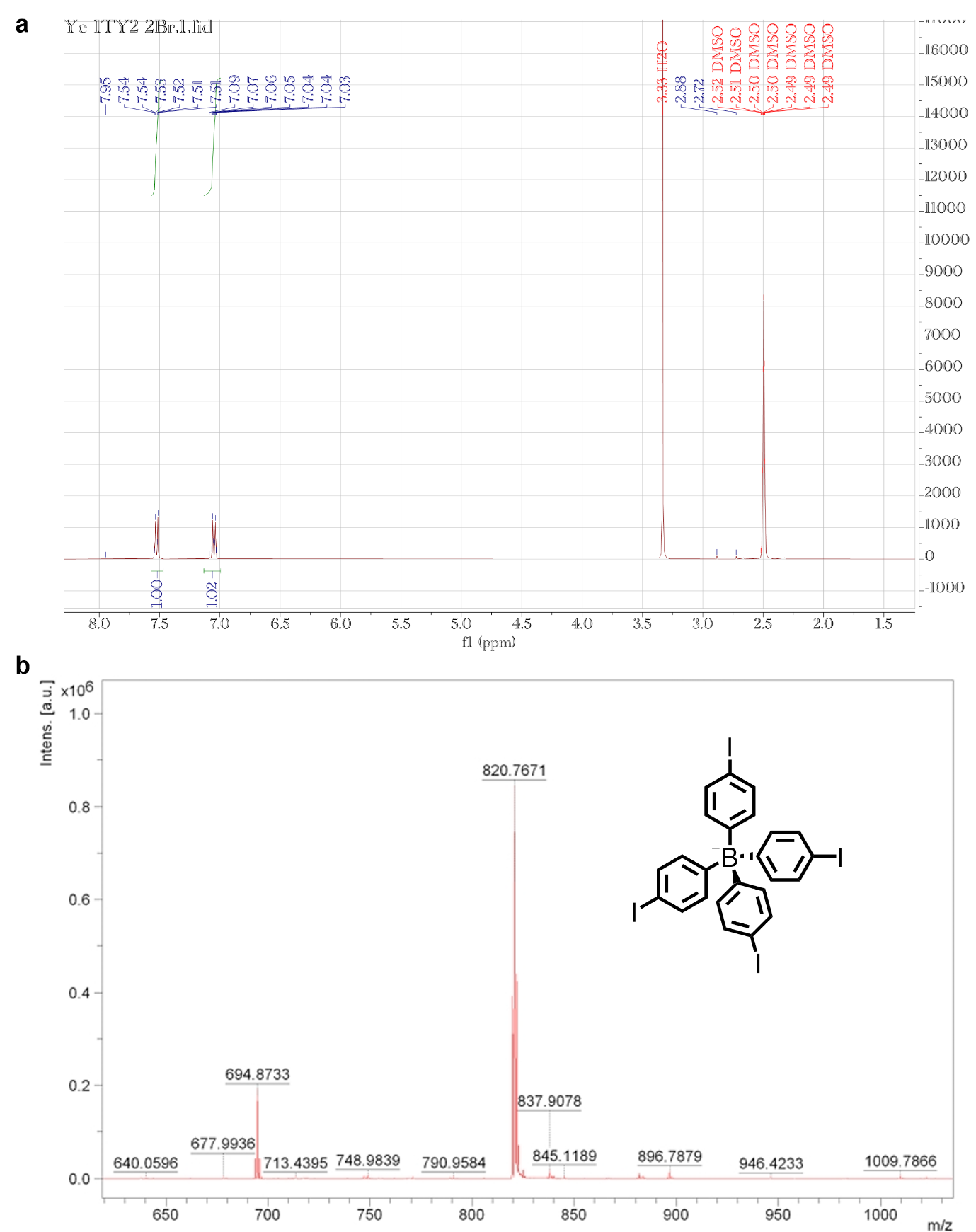


**Figure S1. a)^1^H NMR spectrum (400 MHz, CDCl_3_) of lithium tetrakis(4-iodophenyl)borate. b) Maldi-TOF spectrum of lithium tetrakis(4-iodophenyl)borate.**

**Synthesis of** **Lithium tetrakis(4-formylphenyl)borate**

**Scheme S2. Synthetic procedure of Lithium tetrakis(4-formylphenyl)borate.**

A solution of lithium tetrakis(4-iodophenyl)borate (9.0 g; 10.5 mmol) in dry THF (50 mL) was added to a stirred solution of *n*-BuLi (2.0 M in cyclohexane, 50 mL, 85 mmol) in THF (150 mL) at −78 °C. The resulting dark-green mixture containing the tetralithio intermediate was stirred for 1 h and cooled to −100 °C. It was quenched with DMF (1.8 g; 24.6 mmol). The resulting white suspension was stirred at −78 °C for 30 min, then warmed to r.t., and evaporated under reduced pressure to leave a white solid. It was mixed with Et_2_O (50 mL), and the suspension was filtered, and the solid was washed with hexane (10 mL) and resuspended in hexane (10 mL). Then the mixture was hydrolyzed aq. LiCl (10 wt.%, 10 mL) and aq. HCl (4 M, ca. 5 mL) was added to reach the pH of ca. 3. The obtained yellow slurry was filtered to give a crude product. It was washed with water (2 × 10 mL) and DCM (4 × 5 mL), and dried to give as a cream-white powder, dec. >220 °C, yield: 0.91 g (65%). ^1^H NMR (400 MHz, acetone-d_6_) δ 9.89 (s, 4H), 7.59 (d, J = 7.9 Hz, 8H), 7.53–7.48 (m, 8H) ppm. MS (MALDI-TOF, m/z) calculated for C_28_ H_20_ BO_4_^–^, 431.15; found 429.2946.


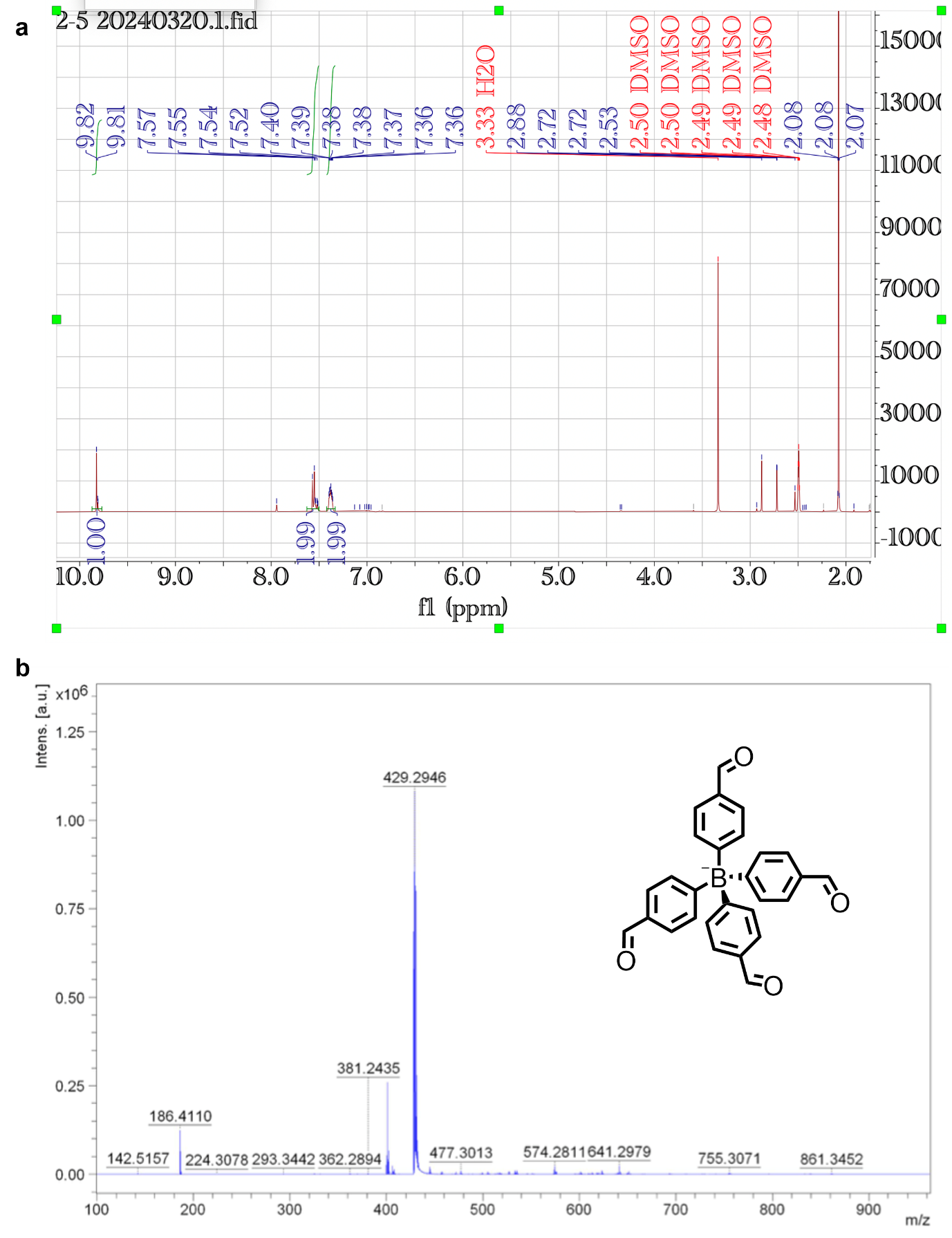


**Figure S2. a)^1^H NMR spectrum (400 MHz, CDCl_3_) of Lithium tetrakis(4-formylphenyl)borate. b) Maldi-TOF spectrum of Lithium tetrakis(4-formylphenyl)borate.**

**Synthesis of borate covalent organic framework (B-COF)**

**Scheme S3. Synthetic procedure of B-COF.**

The lithium tetrakis(4-formylphenyl)borate (20 mg; 0.058 mmol) and *p*-phenylenediamine monomers (12.6 mg; 0.117 mmol) dissolved in a mixed solution of 1,4-dioxane/mesitylene (1 mL/0.1 mL) by sonication (lithium tetrakis(4-formylphenyl)borate and *p*-phenylenediamine for 30 min and 5 min, respectively). Then, two monomer solutions were mixed together with HOAc (6 M, 0.1 mL) in a Schlenk tube (10 mL), and the mixture was degassed via three freeze–pump–thaw cycles. The tube was sealed off and heated at 120 °C for 4 days. The resulting precipitate was collected by vacuum filtration, washed with acetone, ethanol, DMF and subjected to Soxhlet extraction with THF/methanol (1:1, v:v) over one day. The powder was collected with vacuum filtration and dried at 120 °C under vacuum overnight to give B-COFs in yields of 85%. When using aniline as the modulator, it (12 equiv based on the aldehyde group in the reaction system) was added with *p*-phenylenediamine monomer.

**Preparation of Li^+^@B-COFs**

100 mg B-COFs and a stir bar were added to a 20 mL Schlenk flask. A LiTFSI solution (42.85 mg in 2 mL MeOH) was then added and stirred for 1 h. MeOH was removed under vacuum at 70 °C overnight to yield Li^+^@B-COFs.

**Preparation of pellet solid electrolyte**

The vacuum-dried Li^+^@B-COFs, 40 mg, were added to a stainless-steel die (15 mm) and then pressed at ca. 20 MPa for 2 min to yield samples with a thickness of 130 µm. A caliper measured the thickness of the pellet obtained.

**Ionic conductivity (*σ*)**

During the symmetric cell assembly, 5 wt.% PC was dropped on each lithium metal electrode, then the prepared solid electrolyte pellets. Then, placed between symmetrical Li-ion blocking stainless-steel electrodes in a CR2032 coin cell for electrochemical impedance spectroscopy (EIS) measurements over a frequency range of 1 MHz to 0.1 Hz with an amplitude of 10 mV (Autolab PGSTAT204). Measurements were carried out from the highest temperature of 80 °C to r.t. *σ* (mS cm^−1^) was calculated by the following equation (1):


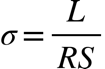
 (1)

where *L* is the thickness of the pellet, *R* is the resistance measured by the EIS, and *S* is the area of SE pellet contact with the electrodes.

The activation energy was calculated from the Arrhenius plot using below equation (2):


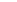

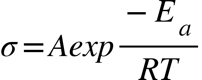
 (2)

where *σ* is the ionic conductivity of the SE pellet at different temperatures, *A* is the pre-exponential factor, *E*_a_ is the pseudo activation energy for Li^+^ transport, *R* is the gas constant, and *T* (K) is the corresponding temperature.

**Li^+^ transference number (*t*_Li+_)**

*t*_Li+_ was calculated by the Bruce−Vincent−Evans (BVE) technique shown as equation (3). The measurement was conducted by Li|Li symmetrical cell with AC impedance and DC polarization process.


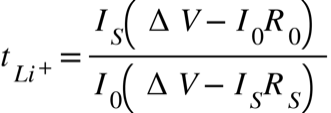
 (3)

where *I*_0_ and *I*_s_ are current at initial and steady-state under ΔV = 10 mV DC polarization, respectively. *R*_0_ with *R*_s_ are the resistance at the initial and steady-state measured by AC impedance.

**Electrochemical stability testing**

Linear sweep voltammetry (LSV) is used to evaluate the oxidation stability of the SE pellets. The LSV is measured from –0.5 V to 5 V *vs*. Li^+^/Li (scan rate of 1 mV s^–1^) with an architecture of stainless-steel|Li^+^@B-COF|Li cell at r.t.

**Preparation of full cells with LiFePO_4_/NCM811 cathodes**

The LiFePO_4_ (LFP)/NCM811 cathode was prepared by mixing LiFePO_4_/NCM81 (80 wt.%), super P (10 wt.%), and PVDF (10 wt.%) with *N*-methyl-2-pyrrolidone to form a slurry and then coated onto Al/C foil and drying at 100 °C for 24 h. The loading of active material is controlled to be around 2.5 mg cm^–2^. The cathode material was dried at 80 °C under vacuum overnight. The areal mass loading of active cathode material on the current collector is 10 mg cm^–2^. Placing the cathode, COFs pellet with 10 wt.% PC between electrolyte and electrodes, and Li metal (200 μm thickness) in a CR2032 coin cell in an Ar-filled glove box (the contents of H_2_O and O_2_ are all below 0.1 ppm) to obtain the final battery. The battery performance was performed by Neware battery test system at the current density of 0.5 C at r.t. with a voltage range of 2.5–4.0 V.

**Materials Characterization data**


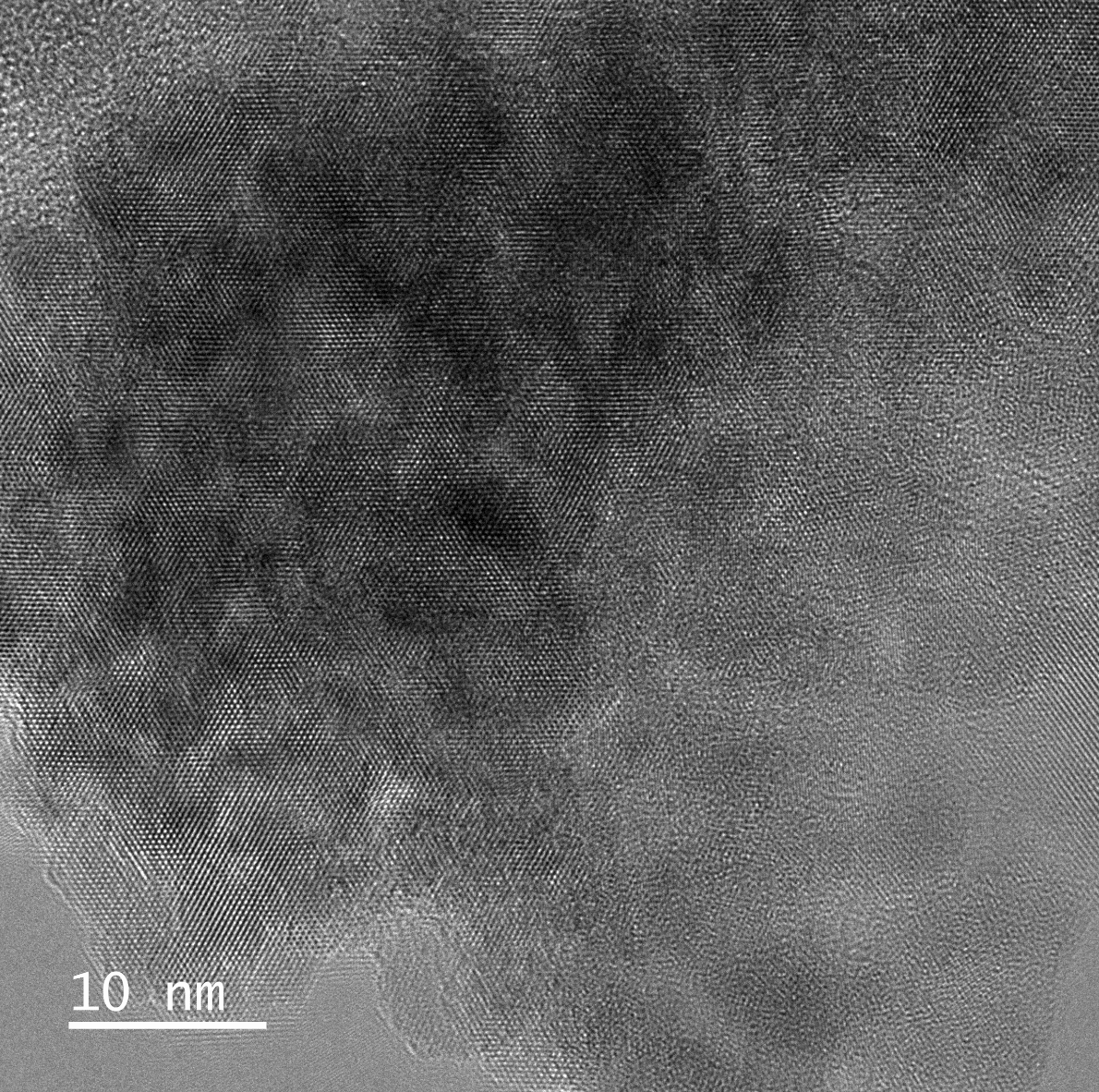


**Figure S3. Large area TEM image of B-COF in Fig. 1g**


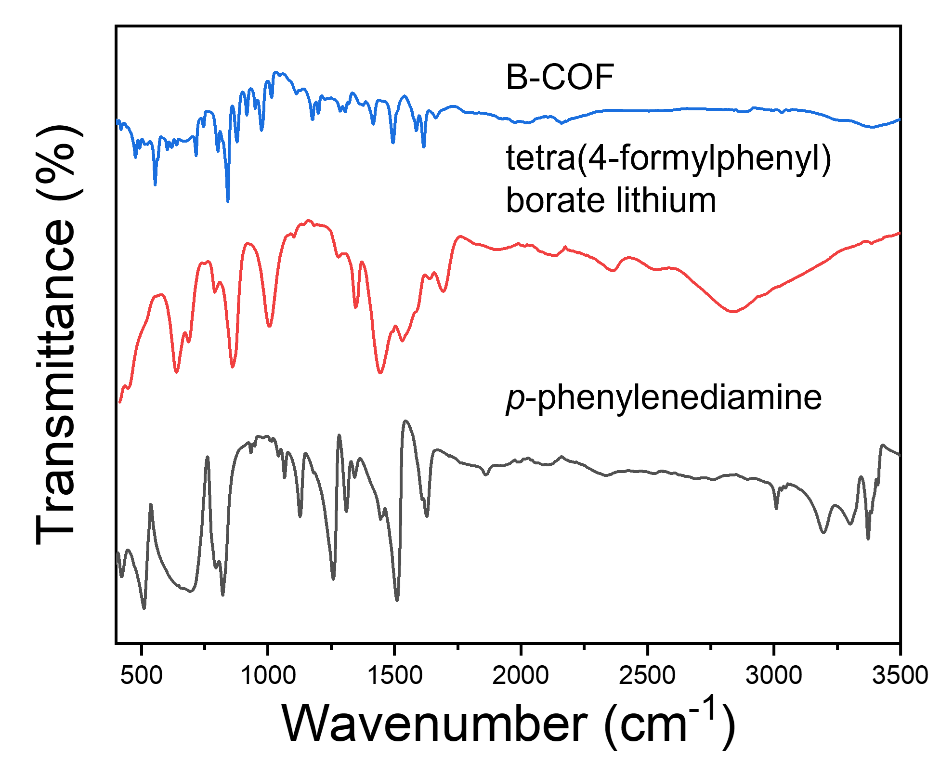


**Figure S4. FTIR spectrum of B-COF, tetra(4-formylphenyl) borate lithium and *p*-phenylenediamine.**

The disappearance of the amine peaks at 3,298 and 3,194 cm^–1^ and the aldehyde peak at 1,696 cm^–1^, along with the emergence of an imine bond stretching vibration peak at 1,614 cm^–1^, indicate the complete conversion of monomers into the imine-based COF.


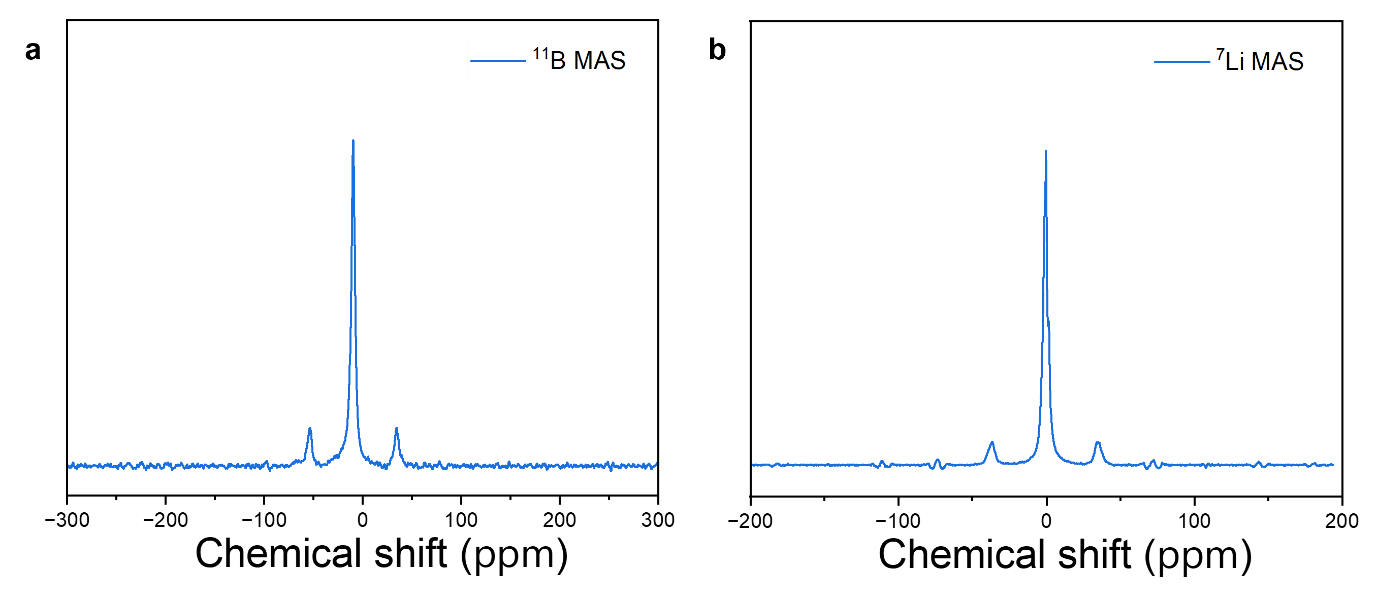


**Figure S5. Solid-state ^11^B (a), and ^7^Li (b) NMR of B-COF.**


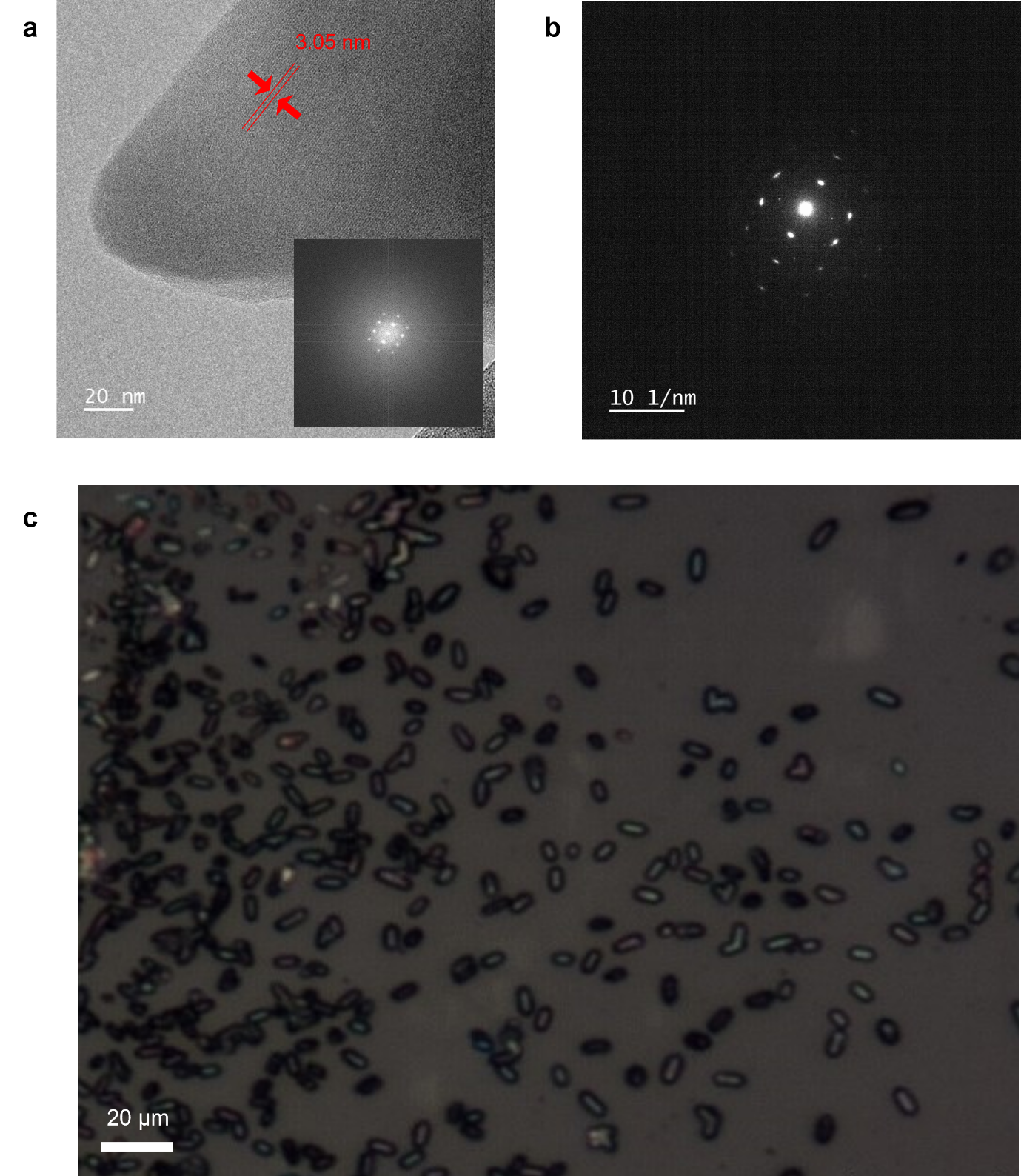


**Figure S6. Morphology of B-COF crystals. a,** High-resolution TEM images of B-COF. The insets show the corresponding FFT patterns. **b,** SAED pattern of (a). **c,** Optical microscopy image of B-COF crystals.


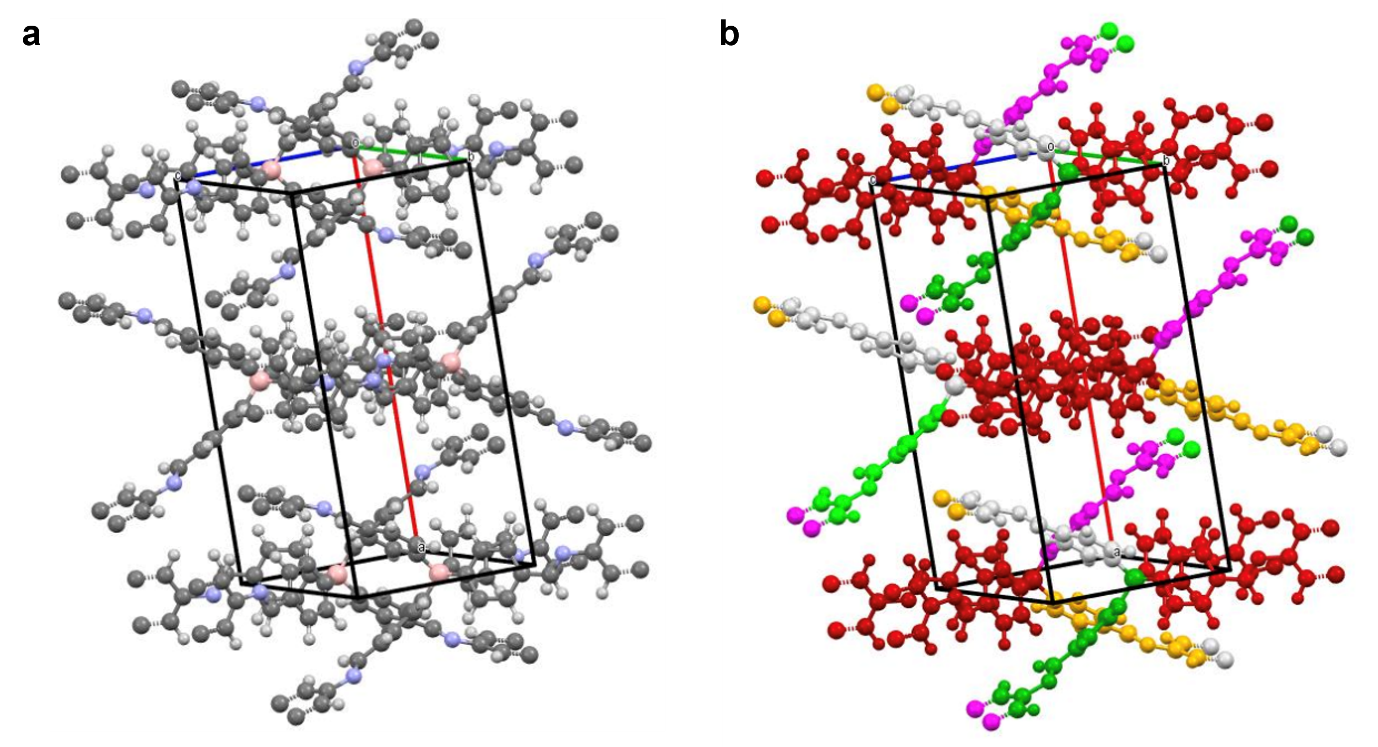


**Figure S7. Crystal cell structure in the B-COF. a,** Colored by atomic category. Gray, blue, light pink, and white, for C, N, B, and H, respectively. **b,** Colored by symmetry relationships.


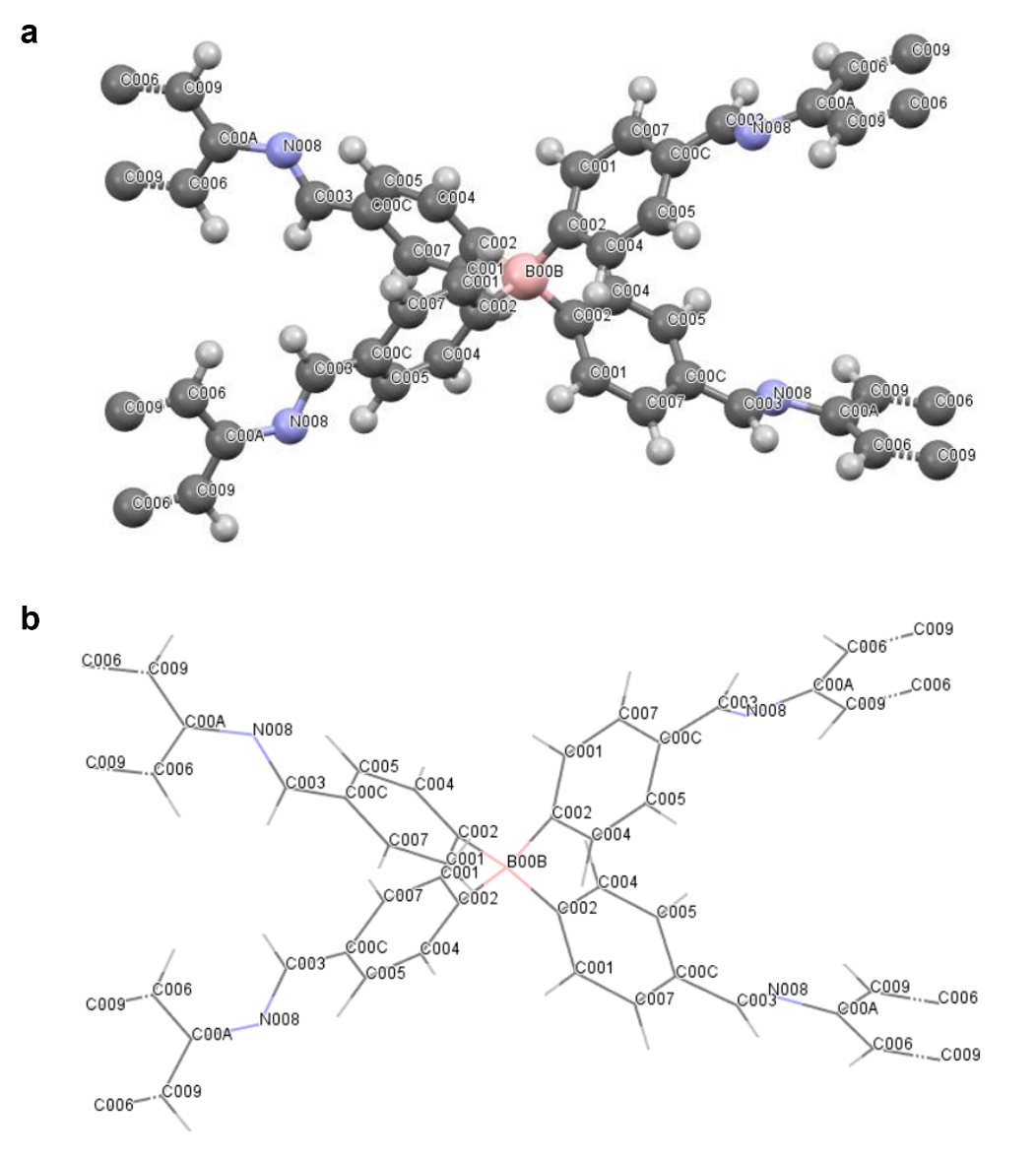


**Figure S8. Molecular structure and atomic labeling in the B-COF crystal. a,** Ball and stick diagram. **b,** Line diagram. Gray, blue, light pink, and white, for C, N, B, and H, respectively.

**Table S1. Fractional Atomic Coordinates (×10^4^) and Equivalent Isotropic Displacement Parameters (Å^2^×10^3^) of the B-COF crystal.**

| Atom | x | y | z | U(eq) |
| --- | --- | --- | --- | --- |
| C001 | 4138(4) | 3210(4) | 189(8) | 18(2) |
| C002 | 4409(4) | 2581(4) | -157(7) | 14.8(19) |
| C003 | 3247(3) | 2869(4) | 3749(7) | 15.0(18) |
| C004 | 4244(3) | 2053(3) | 767(7) | 16(2) |
| C005 | 3846(4) | 2123(4) | 2028(8) | 17.3(19) |
| C006 | 2736(4) | 3067(4) | 6764(8) | 22(2) |
| C007 | 3735(4) | 3292(4) | 1478(8) | 20(2) |
| N008 | 2936(4) | 2394(4) | 4423(9) | 34(2) |
| C009 | 2452(4) | 1908(4) | 6715(9) | 27(2) |
| C00A | 2687(4) | 2468(4) | 5963(9) | 27(2) |
| B00B | 5000 | 2500 | -1250 | 1(3) |
| C00C | 3580(4) | 2741(4) | 2399(8) | 17.0(19) |

**Table S2. Bond Angles.**

| Atom | Atom | Atom | Angle/˚ | Atom | Atom | Atom | Angle/˚ |
| --- | --- | --- | --- | --- | --- | --- | --- |
| C002 | C001 | C007 | 120.2(7) | C009 | C00A | C006 | 117.1(8) |
| C001 | C002 | B00B | 122.0(6) | C009 | C00A | N008 | 119.5(7) |
| C004 | C002 | C001 | 117.1(6) | C0022 | B00B | C0023 | 100.5(5) |
| C004 | C002 | B00B | 119.2(6) | C002 | B00B | C0022 | 114.1(3) |
| N008 | C003 | C00C | 120.2(7) | C002 | B00B | C0024 | 100.5(5) |
| C002 | C004 | C005 | 123.5(7) | C002 | B00B | C0023 | 114.1(3) |
| C00C | C005 | C004 | 120.3(7) | C0024 | B00B | C0023 | 114.1(3) |
| C00A | C006 | C0091 | 120.7(7) | C0022 | B00B | C0024 | 114.1(3) |
| C00C | C007 | C001 | 121.2(7) | C005 | C00C | C003 | 123.5(7) |
| C003 | N008 | C00A | 122.2(7) | C005 | C00C | C007 | 117.7(7) |
| C00A | C009 | C0061 | 122.2(8) | C007 | C00C | C003 | 118.2(7) |
| C006 | C00A | N008 | 123.1(7) |  |  |  |  |

**Table S3. Bond Lengths.**

| Atom | Atom | Length/Å | Atom | Atom | Length/Å |
| --- | --- | --- | --- | --- | --- |
| C001 | C002 | 1.376(10) | C005 | C00C | 1.361(11) |
| C001 | C007 | 1.404(10) | C006 | C0091 | 1.405(11) |
| C002 | C004 | 1.361(9) | C006 | C00A | 1.377(11) |
| C002 | B00B | 1.523(7) | C007 | C00C | 1.390(10) |
| C003 | N008 | 1.265(10) | N008 | C00A | 1.463(12) |
| C003 | C00C | 1.390(10) | C009 | C00A | 1.366(11) |
| C004 | C005 | 1.375(10) |  |  |  |

**Table S4. Torsion Angles.**

| A | B | C | D | Angle/˚ |
| --- | --- | --- | --- | --- |
| C001 | C002 | C004 | C005 | -2.0(11) |
| C001 | C002 | B00B | C0021 | -137.2(8) |
| C001 | C002 | B00B | C0022 | -22.4(6) |
| C001 | C002 | B00B | C0023 | 100.2(7) |
| C001 | C007 | C00C | C003 | 172.5(7) |
| C001 | C007 | C00C | C005 | 1.5(11) |
| C002 | C001 | C007 | C00C | -2.3(11) |
| C002 | C004 | C005 | C00C | 1.3(11) |
| C003 | N008 | C00A | C006 | 3.8(13) |
| C003 | N008 | C00A | C009 | -169.7(8) |
| C004 | C002 | B00B | C0021 | 58.4(4) |
| C004 | C002 | B00B | C0023 | -64.2(5) |
| C004 | C002 | B00B | C0022 | 173.2(6) |
| C004 | C005 | C00C | C003 | -171.5(7) |
| C004 | C005 | C00C | C007 | -1.0(11) |
| C0064 | C009 | C00A | C006 | 0.3(13) |
| C0064 | C009 | C00A | N008 | 174.2(7) |
| C007 | C001 | C002 | C004 | 2.5(11) |
| C007 | C001 | C002 | B00B | -162.2(5) |
| N008 | C003 | C00C | C005 | -28.6(11) |
| N008 | C003 | C00C | C007 | 161.0(7) |
| C0094 | C006 | C00A | N008 | -173.9(7) |
| C0094 | C006 | C00A | C009 | -0.3(12) |
| B00B | C002 | C004 | C005 | 163.1(6) |
| C00C | C003 | N008 | C00A | 168.5(7) |

**Table S5. The two individual datasets of the B-COF crystal were collected using MicroED technology.**

| Data | a/Å b/Å c/Å | α/° | β/° | γ/° | CC1/2 Compl./% | I/σ | Ntotal | Nunique | Rmeas |
| --- | --- | --- | --- | --- | --- | --- | --- | --- | --- |
| Sample 1 | 20.15 20.15 8.87 | 90 | 90 | 90 | 99.2* 0.86 | 2.9 | 4515 | 1745 | 0.20 |
| Sample 2 | 20.29 20.29 8.86 | 90 | 90 | 90 | 99.4* 0.87 | 4.18 | 4219 | 1784 | 0.15 |

**Table S6. Crystallographic and refinement information of the B-COF crystal**

| Identification code | | B-COF |
| --- | --- | --- |
| Empirical formula | | C40H28BN4 |
| Formula weight | | 575.5 |
| Temperature/K | | 77 |
| Crystal system | | tetragonal |
| Space group | | I4_1_/a |
| a/Å | | 19.611(3) |
| b/Å | | 19.611(3) |
| c/Å | | 8.9056(18) |
| α/° | | 90 |
| β/° | | 90 |
| γ/° | | 90 |
| Volume/Å^3^ | | 3424.9(12) |
| Z | | 4 |
| ρcalc g/cm3 | | 1.116 |
| Radiation | | Electron(λ = 0.02508) |
| 2Θ range for data collection/° 0.146 to 1.64  Index ranges -22 ≤ h ≤21, -22 ≤ k ≤22, -9 ≤ l ≤ 9  Reflections collected 6554  Independent reflections 1299 [*R_int_* = 0.1662, *R_sigma_* = 0.1156]  Data/restraints/parameters 1299/0/47 | | |
| Goodness-of-fit on F^2^ | 1.568 | |
| Final R indexes [I>=2σ (I)] | *R_1_* = 0.1674, *wR_2_* = 0.4341 | |
| Final R indexes [all data] | *R_1_* = 0.1989, *wR_2_* = 0.4551 | |


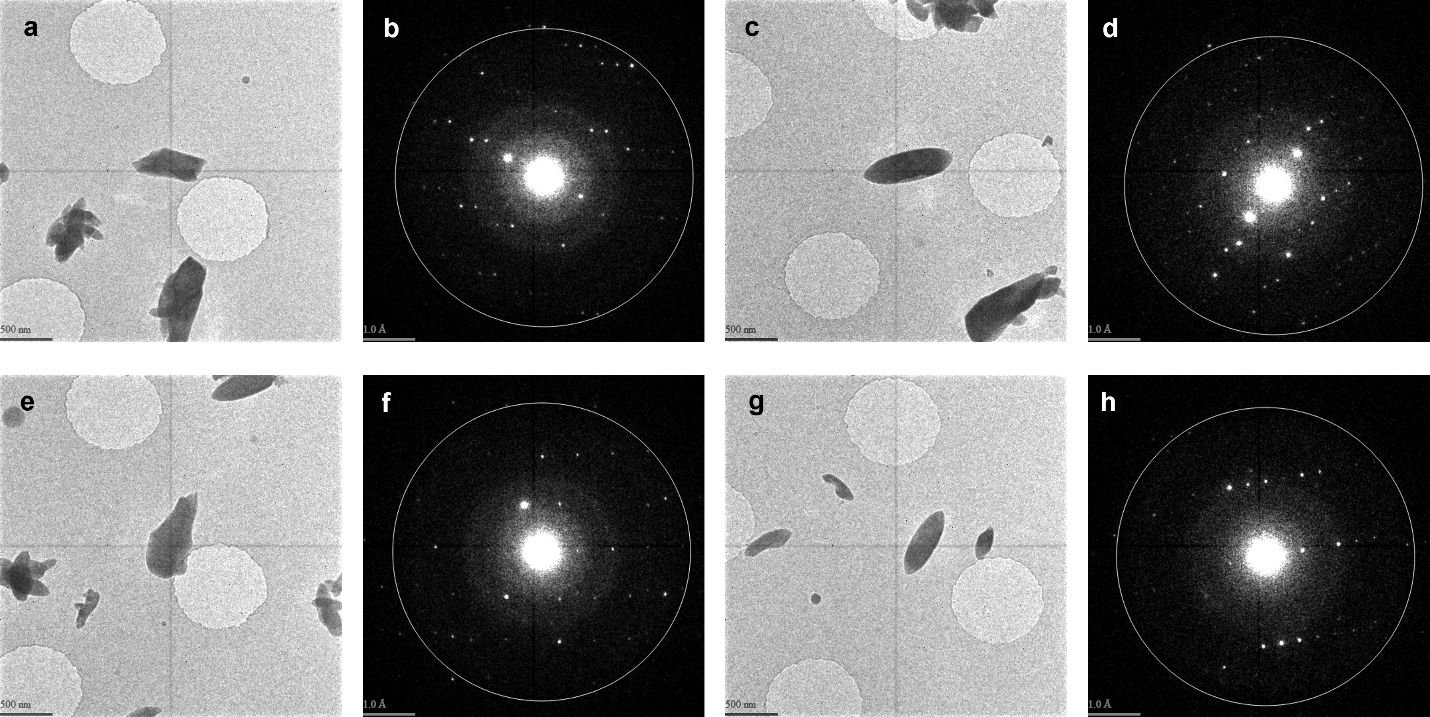


**Figure S9. Crystal TEM images and electron diffraction patterns. a, b,** B-COF crystal sample 1**. c, d,** B-COF crystal sample 2**. e, f,** B-COF crystal sample 3**. g, h,** B-COF crystal sample 4.

**
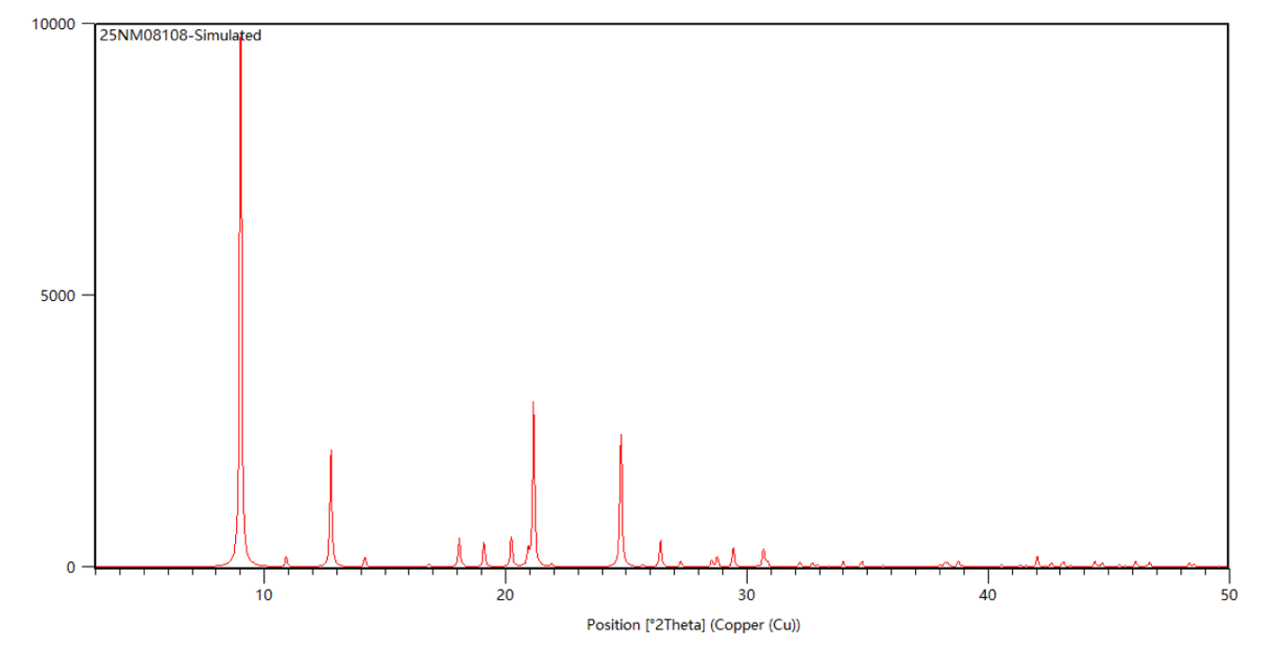
**

**Figure S10. Simulated XRD patterns, based on the crystalline structures of the B-COF single crystals.** This simulated XRD is nearly the same as the experimental PXRD in Fig. 1d.


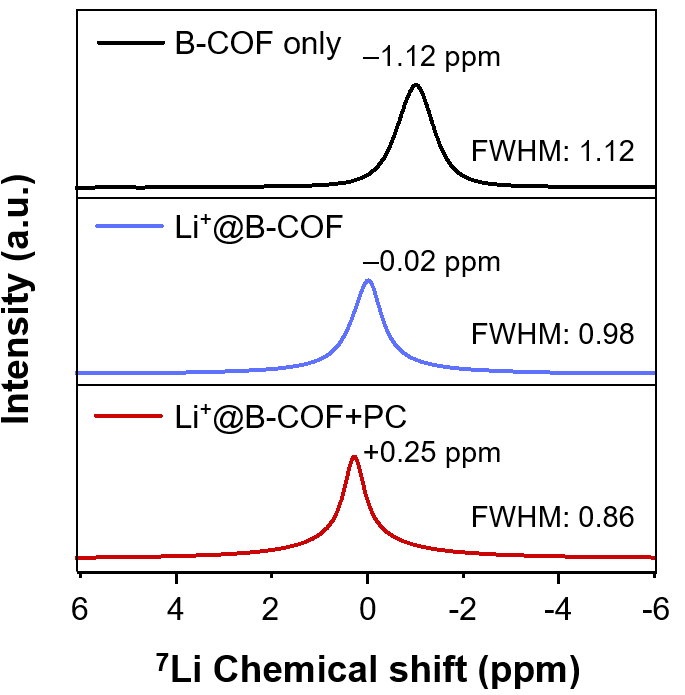


**Figure S11. ^7^Li NMR of the samples, B-COF, Li^+^@B-COF, and Li^+^@B-COF+PC.**


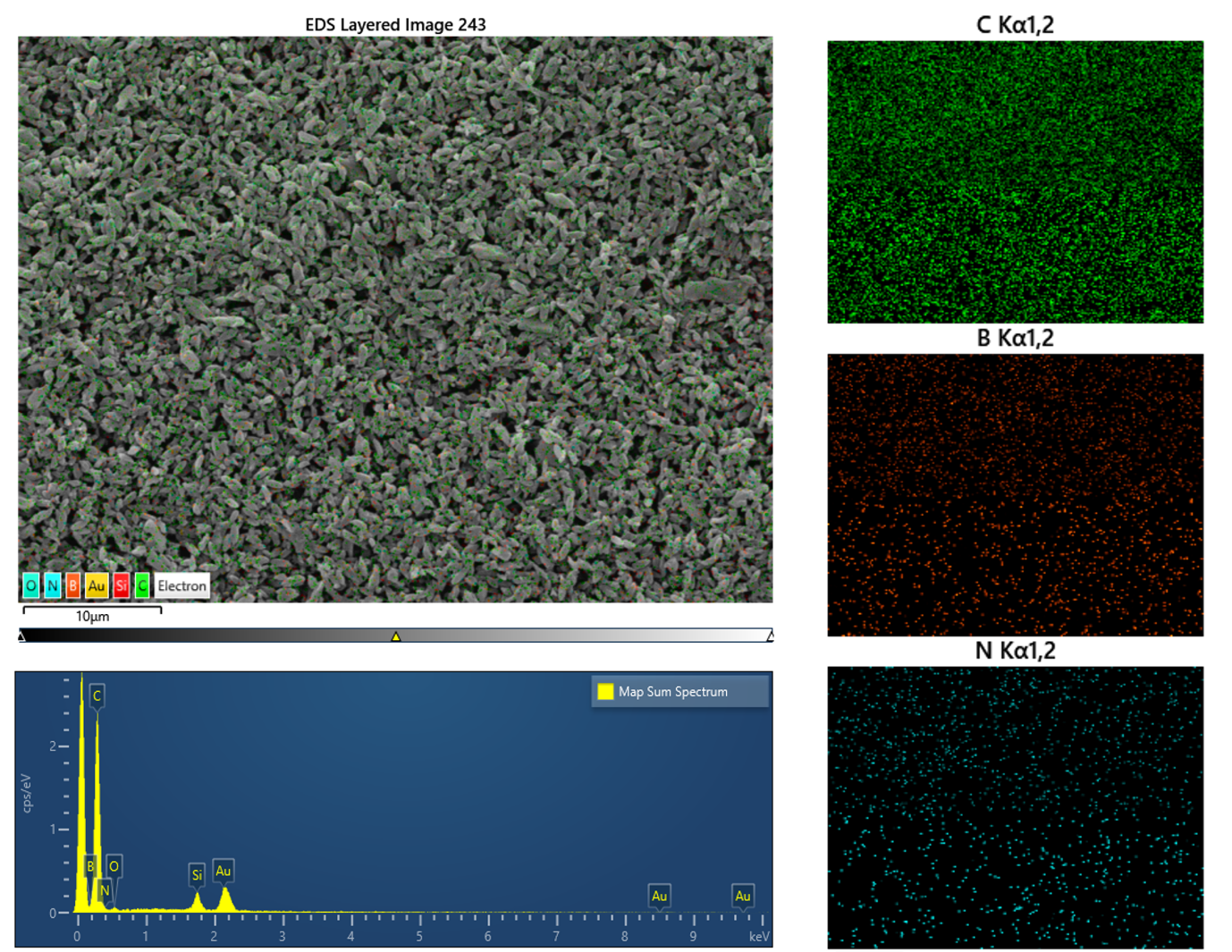


**Figure S12. SEM EDX image of B-COF with distribution images of carbon, boron, and nitrogen.**


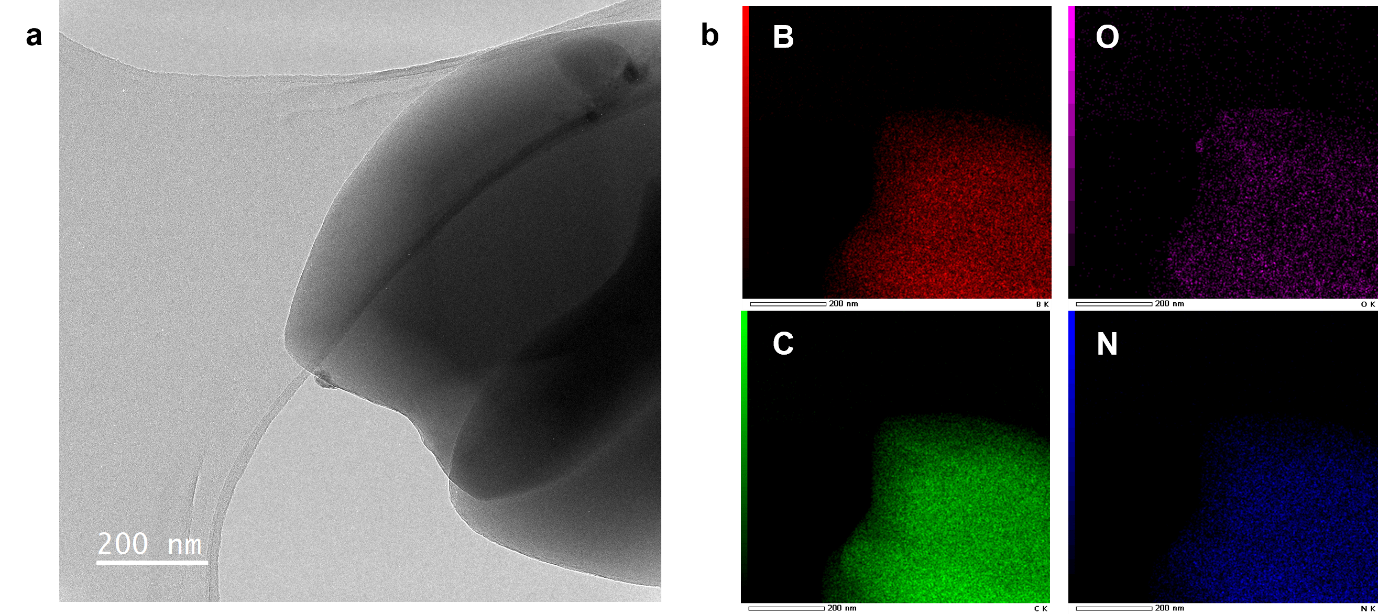


**Figure S13. TEM EDX image of B-COF with distribution images of boron, oxygen, carbon, and nitrogen.**

**Table S7. Inductively Coupled Plasma Optical Emission Spectrometry data and calculated theoretical B and Li element content.**

| Peak | Theoretical value wt.% | Experimental value wt.% |
| --- | --- | --- |
| Li | 1.132% | 1.098% |
| B | 1.780% | 1.752% |


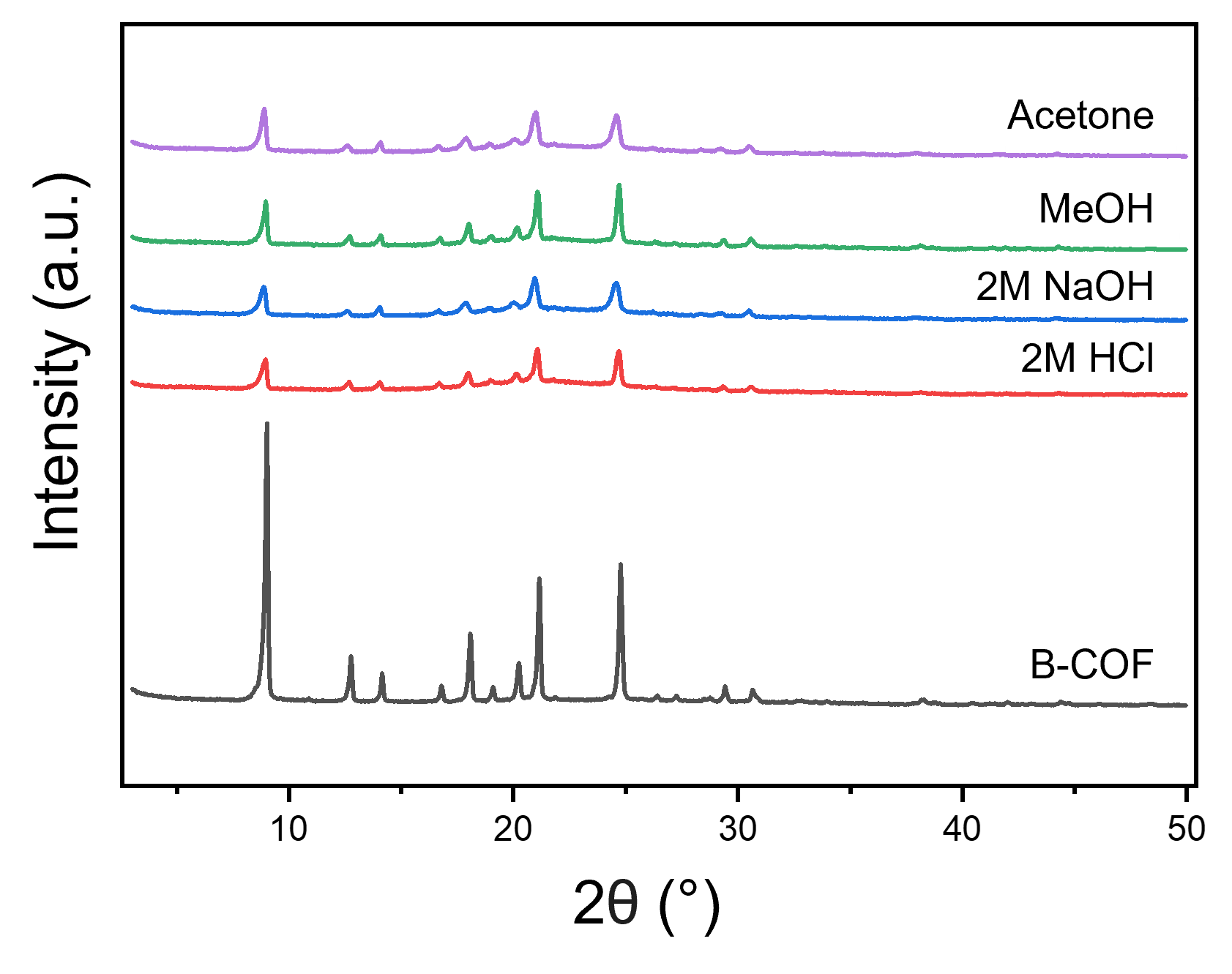


**Figure S14. Experimental PXRD data of B-COF in various solvent conditions.**

Peaks at 8.9, 12.8, 14.1, 15.9, 18.1, 19.0, 21.1, and 25.2°, corresponding to the 200, 220, 211, 301, 400, 231, 420, and 501 reflection planes, respectively.

**Figure S15. Thermogravimetric curves of B-COF crystals.**


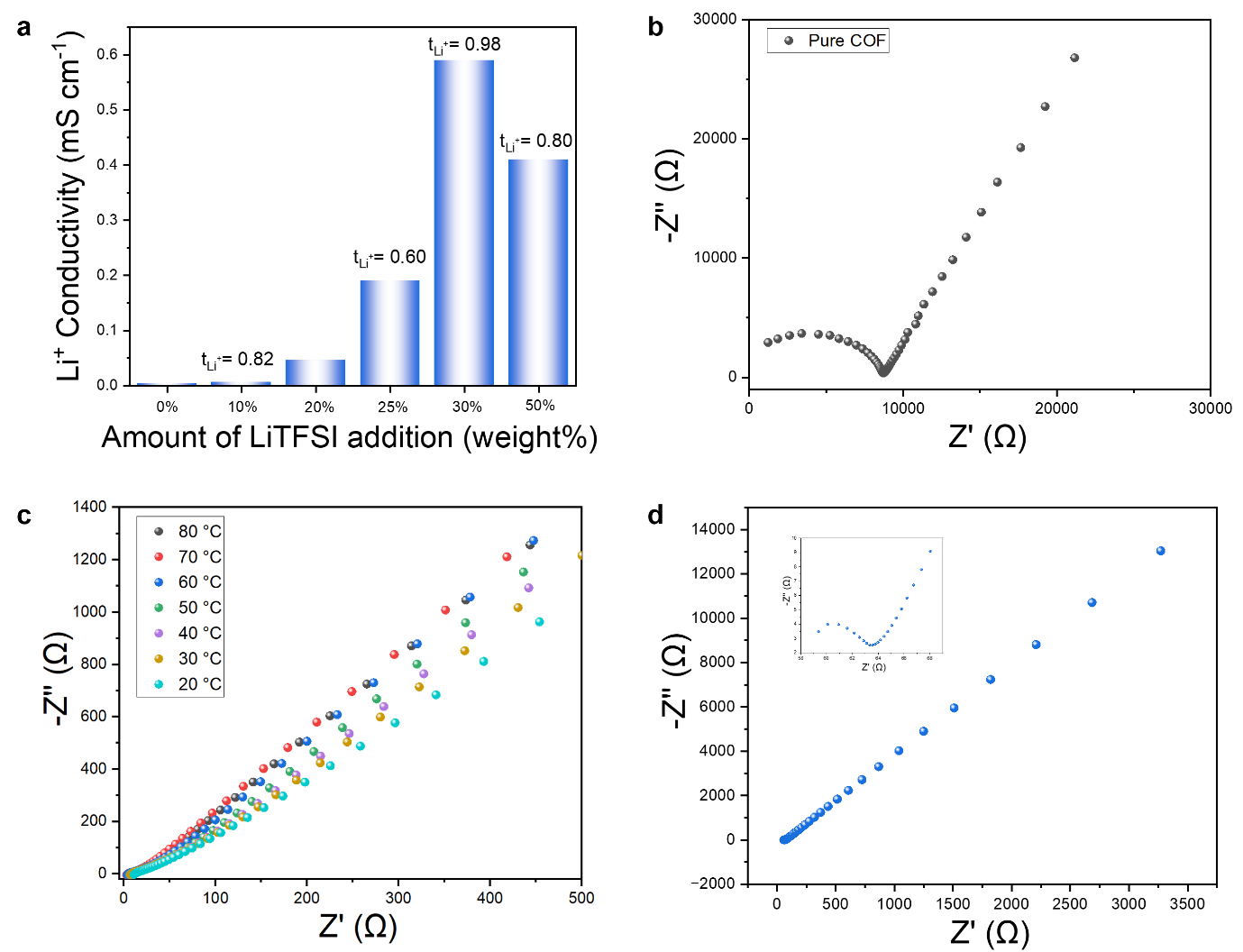


**Figure S16. *σ* and *t*_Li+_ of B-COFs. a,** Li^+^ conductivity and *t*_Li+_ with various amounts of LiTFSI. **b,** Nyquist plot of pure B-COF electrolyte. **c,** Nyquist plot of Li^+^@B-COF electrolyte (30% Li salt added) under different temperatures. **d,** Nyquist plot for Li^+^@B-COF electrolyte with 10 wt.% PC.


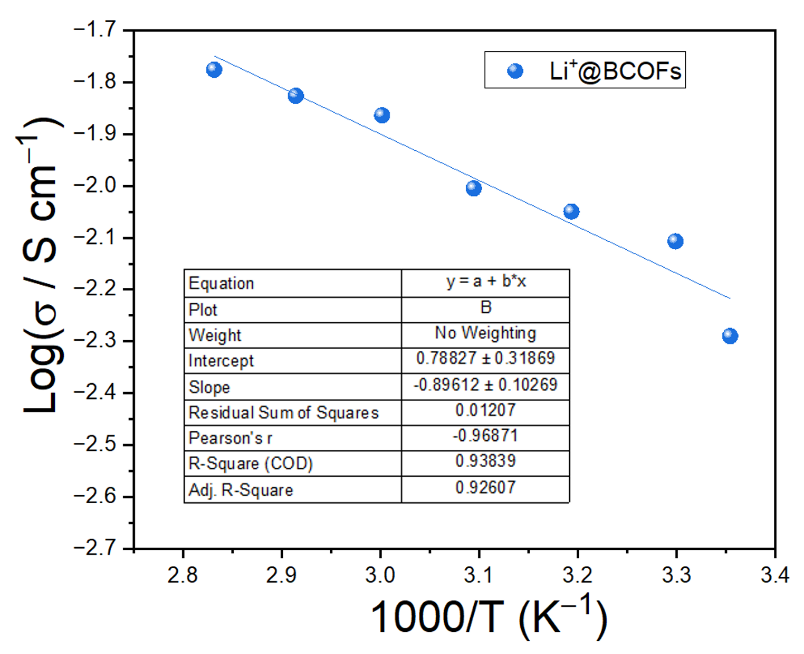


**Figure S17. Arrhenius plot from Li^+^@B-COF.**


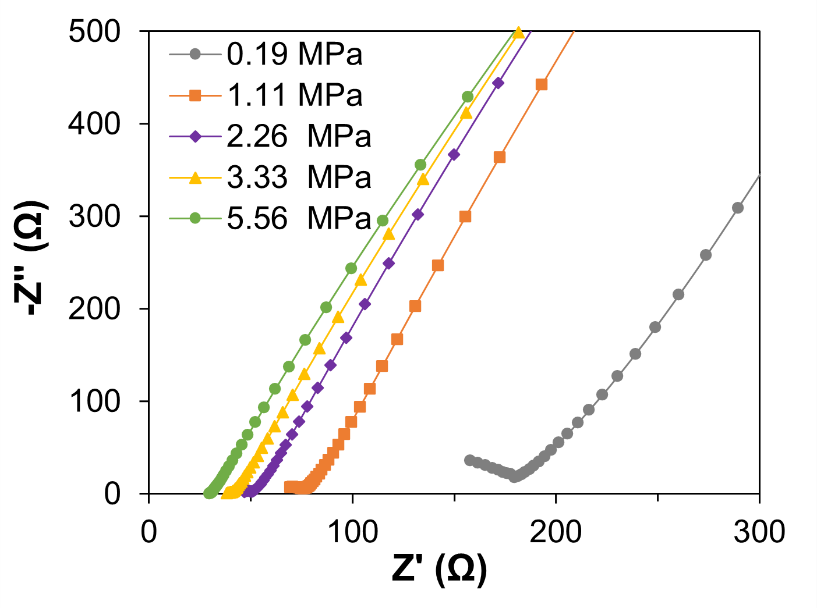


**Figure S18. Nyquist plots from pure B-COFs under various pressures.**


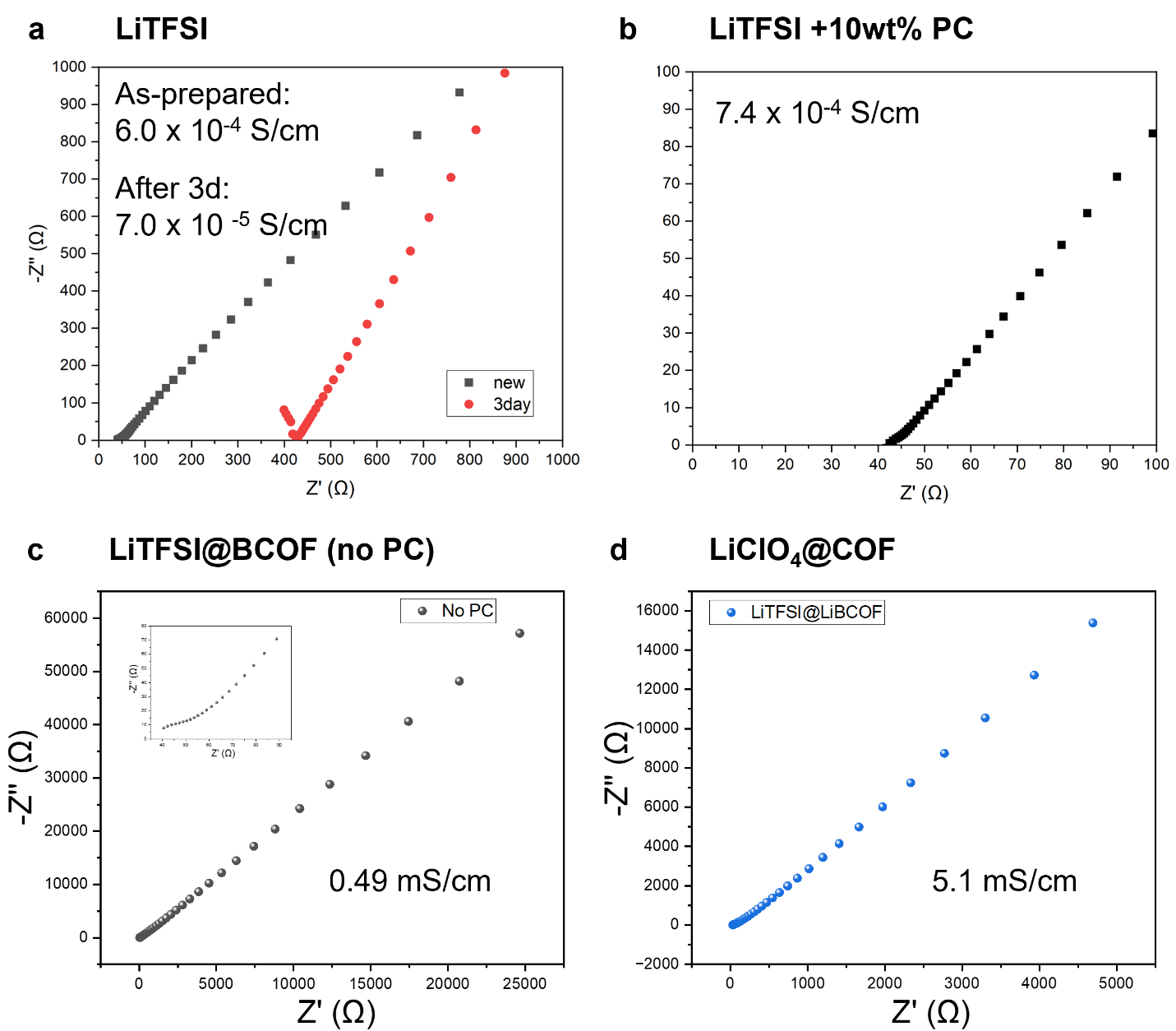


**Figure S19. Ion conductivity of B-COFs with different additives. a,** Nyquist plot and Li^+^ conductivity value for Pure LiTFSI. **b,** Nyquist plot and Li^+^ conductivity value for LiTFSI + PC. **c,** Nyquist plot and Li^+^ conductivity value for LiTFSI + B-COF. **d,** Nyquist plot and Li^+^ conductivity value for LiClO_4_@B-COF.


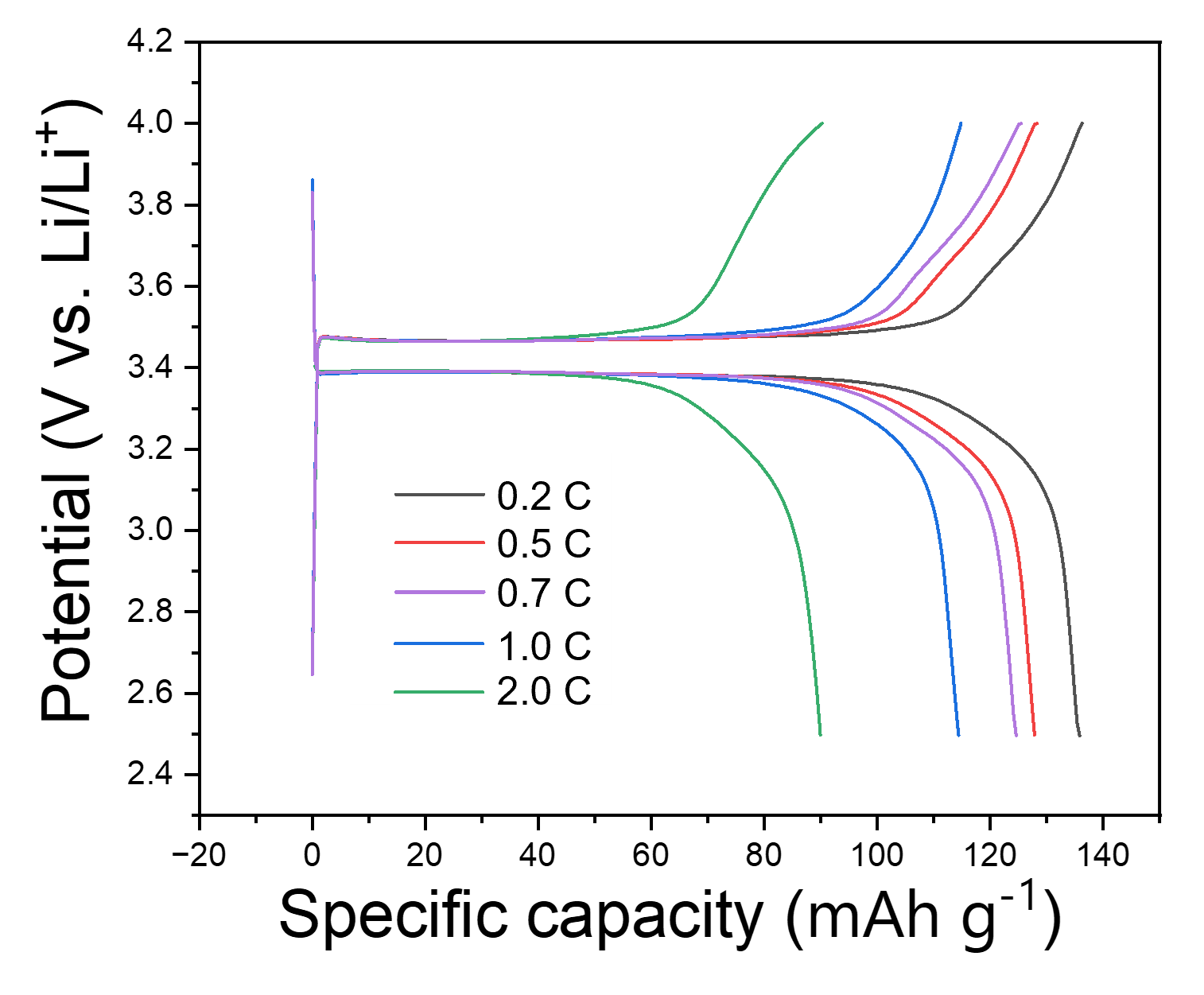


**Figure S20. Charge and discharge curves from a full cell with an LFP cathode at different C rates.**


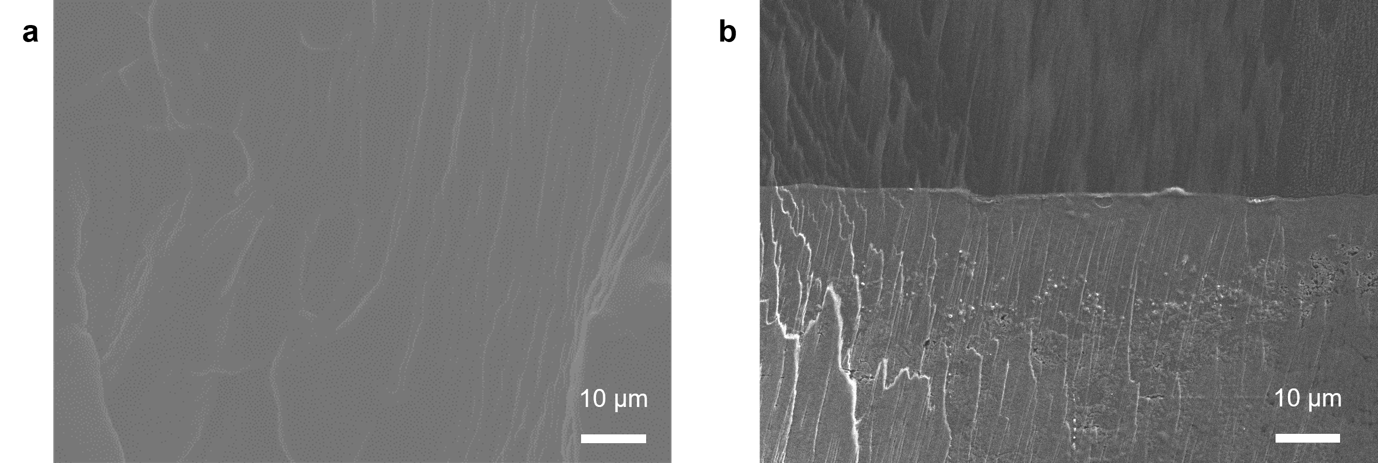


**Figure S21. Post-mortem analysis of the cells. a SEM image of the surface morphology of Li metal’s surface from the** cycled full cell. **b,** SEM image of cross-section interface between B-COF electrolyte and Lithium metal anode after cycling test in a symmetric cell.

We employed an ion milling technique to remove the outer layers of the samples and create cross-sections for unobstructed observation. Within an argon atmosphere protection system, the battery cross-section samples were shielded and positioned in the path of a broad argon ion beam. By carefully placing the samples at the desired cross-sectional locations, unwanted rough sections were cut away by the ion beam, resulting in new cross-sections suitable for SEM analysis. We polished the cross-section of the lithium metal electrode from the cycled symmetric battery and obtained clear images of the electrode-electrolyte interface.


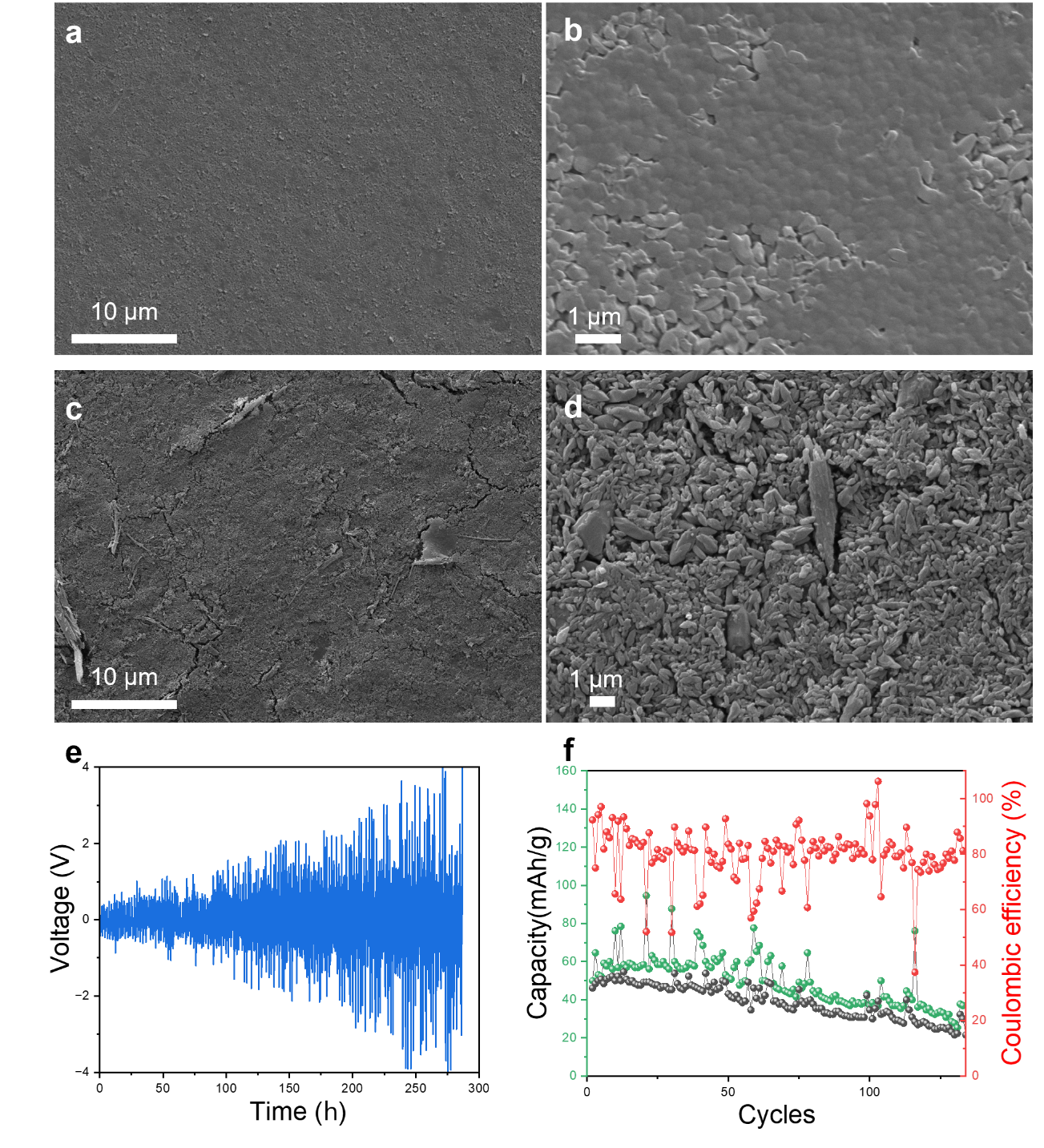


**Figure S22. SEM image of different grain boundary electrolyte pellets and its impact on cell performance a, b,** SEM image of small crystal B-COF sample electrolyte pellet surface. **c, d,** SEM image of uneven crystal B-COF sample electrolyte pellet surface. The average crystal length is 4.5 µm. **e,** Galvanostatic cycling at a current density of 0.1 mA cm^–2^ in Li||Li symmetric cells with non-homogeneous B-COF crystals. **f,** Cycling in Li||LFP cells charged to 4.2 V with non-homogeneous crystal B-COF crystals at 0.5 C.

To synthesize larger crystals, 12 equiv of aniline as the modulator, based on the aldehyde group in the reaction system, was added with *p*-phenylenediamine monomer.


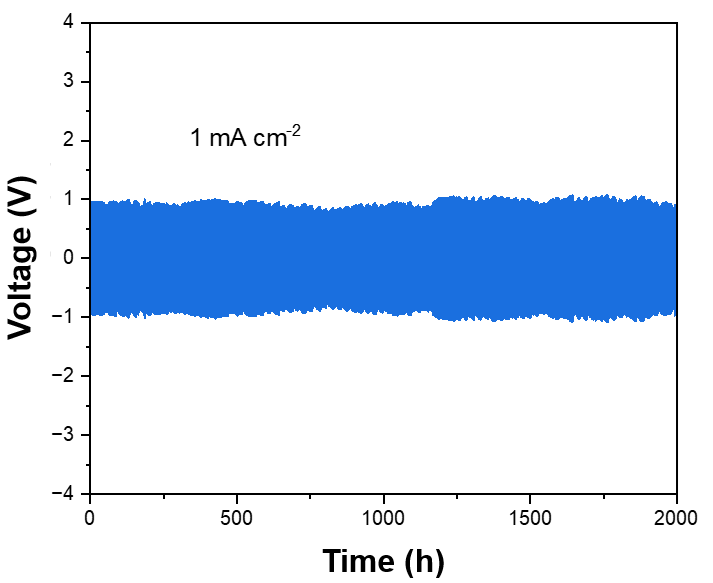


**Figure S23. Galvanostatic cycling at a current density of 1 mA cm^–2^ in Li||Li symmetric cells with Li^+^@B-COF.**

**Figure S24. The voltage profiles for plating 15 µm lithium at the Li|Li^+^@B-COF interface for electron backscatter diffraction characterization.**

The lithium film is deposited at a current density of 200 µA cm^–2^ within the Li|Li^+^@B-COF|Li symmetric battery configuration. the voltage distribution during the Li deposition process initially registered at 50 mV, subsequently stabilizing around 20 mV. The elevated initial voltage indicates a crystal nucleation phase, which ensures uniform layer growth. Following a deposition of 3 mAh cm^–2^, the battery is disassembled in an inert glove box to prevent air exposure and transferred for ion milling treatment to prepare cross-sectional samples.


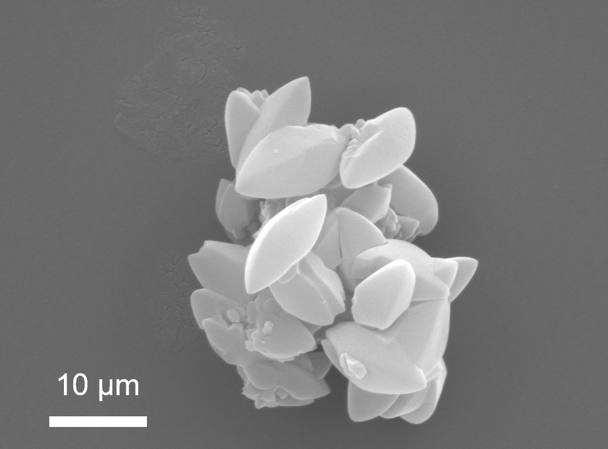


**Figure S25. SEM image of B-COF crystal particles after pressing them at 2.26 MPa for 1 month.**


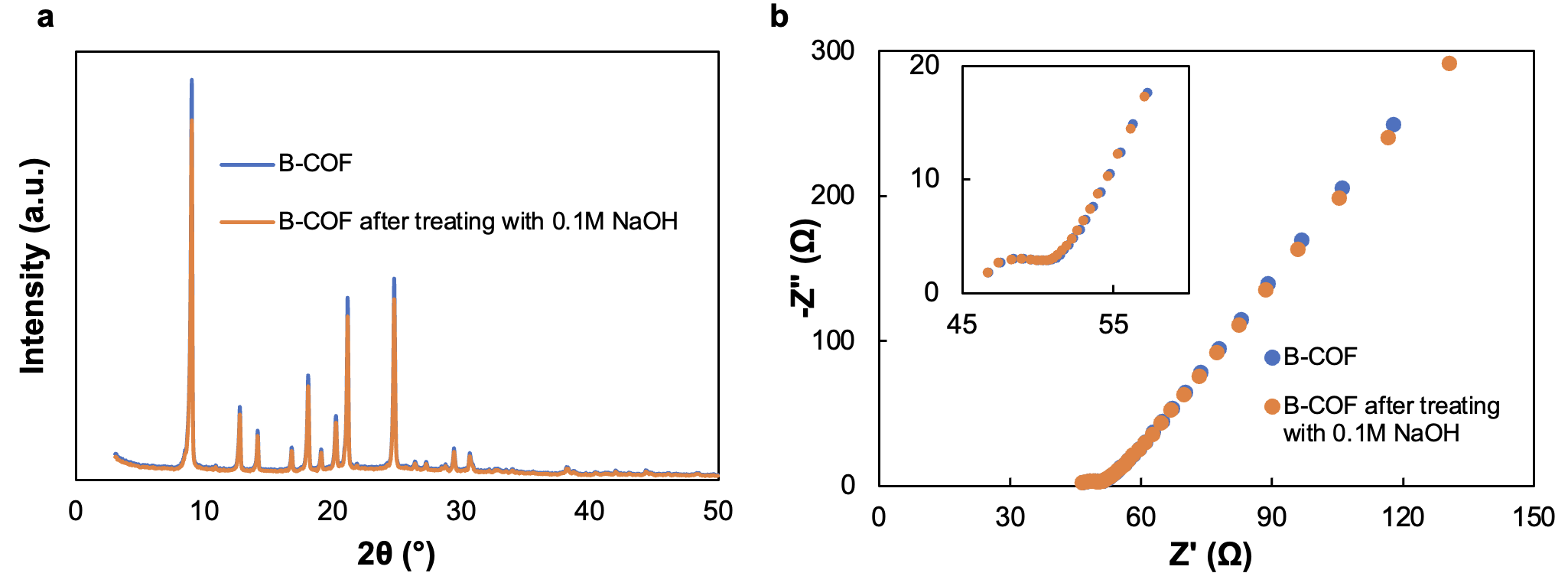


**Figure S26. Post-stability tests of B-COF samples after treating them with 0.1 M NaOH solution for one month.**

**Table S8. Recently published COF-based electrolyte performance.**

| **Sample names** | **Ionic centers** | **Additives** | ***σ* (mS cm^–1^)** | ***t*_Li+_** | **Ea (eV)** | **Ref.** |
| --- | --- | --- | --- | --- | --- | --- |
| **B-COF** | **Borate** | **LiTFSI, 10 wt.% PC** | **8.10** | **0.98** | **0.07** | **This work** |
| ICOF-2:PVDF | Spiroborate | PVDF/PC | 3.1E-02 | 0.8 | 0.24 | ^1^ |
| PEG-Li^+^@CD-COF-Li | Spiroborate | LiClO_4_/PEG-800 | 2.6E-02 | 0.2 | 0.17 | ^2^ |
| TpPa-SO_3_Li | Sulfonate | N/A | 2.7E-02 | 0.9 | 0.18 | ^3^ |
| H-Li-ImCOF | Imidazolate | 20 wt.% PC | 5.3 | 0.88 | 0.12 | ^4^ |
| CH_3_-Li-ImCOF | Imidazolate | 20 wt.% PC | 8.0E-02 | 0.93 | 0.27 | ^4^ |
| CF_3_-Li-ImCOF | Imidazolate | 20 wt.% PC | 7.2 | 0.81 | 0.1 | ^4^ |
| LiPF_6_@Ge-COF-1 | Germanate | 20 wt.% LiPF_6_/EC-DEC | 2.5E-01 | 0.67 | 0.29 | ^5^ |
| LiCON-1 | Phenolate | N/A | 2.1E-04 | 0.86 | 0.25 | ^6^ |
| LiCON-2 | Phenolate, Carboxylate | N/A | 4.4E-03 | 0.83 | 0.22 | ^6^ |
| LiCON-3 | Phenolate, Sulfonate | N/A | 3.2E-02 | 0.92 | 0.13 | ^6^ |
| LE@ACOF | Silicate | LiPF_6_/EC-DEC | 3.7 | 0.82 | 0.15 | ^7^ |
| ﻿Li-CON-TFSI | Guanidinium | LiTFSI | 5.7E-02 | 0.61 | 0.34 | ^8^ |
| PEG-Li^+^@EB-COF-ClO_4_ | ﻿Ethidium | LiClO_4_/PEG-800 | 1.9E-02 | 0.6 | 0.21 | ^2^ |
| Im-COF-TFSI@Li | Imidazolium | LiTFSI | 2.9E-02 | 0.62 | 0.32 | ^9^ |
| ﻿dCOF-ImTFSI-60@Li | Imidazolium | LiTFSI | 9.7E-02 | 0.72 | 0.28 | ^9^ |
| PEG-Li^+^@COF-300 | NA | LiClO_4_/PEG-800 | 1.4E-03 | 0.44 | 0.2 | ^2^ |
| PEG-Li^+^@COF-5 | NA | LiClO_4_/PEG-800 | 3.6E-05 | 0.4 | 0.35 | ^2^ |
| H-ImCOF@LiClO_4_ | NA | LiClO_4_/20 wt.% PC | 4.0E-02 | 0.207 | 0.21 | ^4^ |

**Computational studies of the Li^+^ transport**

Based on our preliminary calculation results (Table S9), we obtained the binding energies between Li⁺ and B-COF, as well as between Li⁺ and COF-303 (Charge neutral counterpart of B-COF; Please see *Science* 361, 48–52 (2018) for more information about the COF-303). The binding energy of Li⁺ with B-COF is –6.047 eV, which is significantly stronger than that with COF-303. The binding energy between Li⁺ and TFSI^－^ is calculated to be –5.379 eV, which is weaker than the interaction between Li⁺ and B-COF but stronger than that between Li⁺ and COF-303. This suggests that B-COF has a significantly greater capacity to dissociate LiTFSI than COF-303, thereby facilitating more efficient Li⁺ transport. Consequently, this enhanced dissociation effect is identified as the primary mechanism underlying the superior ion-transport performance observed in the B-COF system (Fig. S27).

Furthermore, given the strong binding energy, which could lead to Li⁺ trapping at boron (B) sites, we calculated diffusion energy barriers along plausible transport pathways to ensure high ion mobility. We employed density functional theory (DFT) as a rigorous and practical approach to calculate the Li⁺ migration barriers within the COFs. We constructed representative symmetric structural units for both COFs. Subsequently, the Climbing Image Nudged Elastic Band (CI-NEB) method was employed to evaluate the energy barriers for Li⁺ migration from the nitrogen (N) site to the central atom (boron for B-COF or carbon for COF-303). Given the structural symmetry, the reverse process, moving away from the central atom, was considered the inverse of the illustrated pathway. As shown in Fig. 5, the Li⁺ diffusion in the B-COF system exhibited a two-step hopping mechanism with energy barriers of 0.370–0.380 eV and 0.220–0.357 eV for each step, respectively. These values are comparable to the single-step diffusion barrier observed in C-COF-303 (0.341–0.395 eV). Therefore, while the B-COF system promotes strong dissociation of LiTFSI, the B center, once coordinated with intrinsic Li⁺, does not significantly impede the hopping transport of additional Li⁺ introduced by LiTFSI.


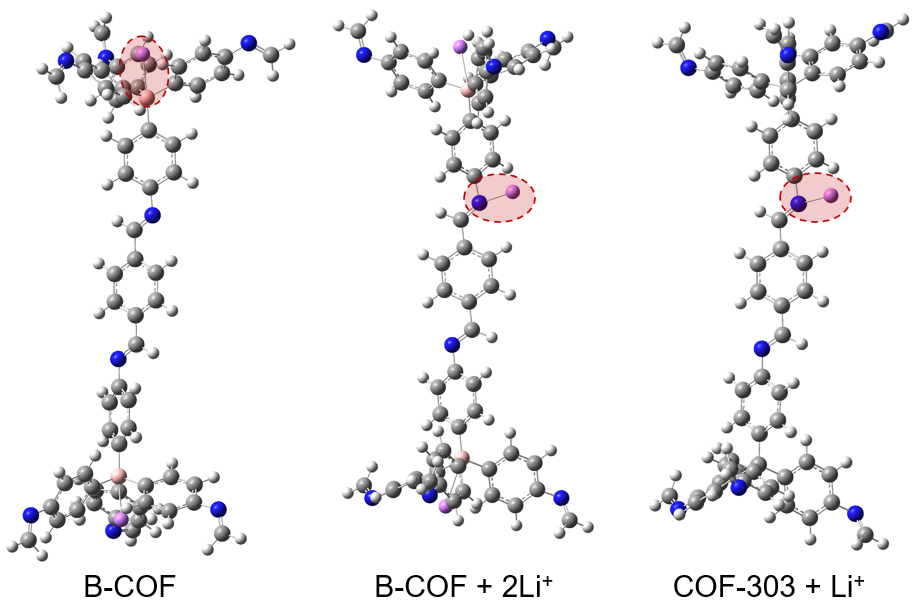


**Figure S27. Model structures of pure B-COF, B-COF + 2Li^+^ (with excess Li^+^), and COF-303 + Li^+^ (charge-neutral COFs with one excess Li^+^).**

**Table S9. Summarized property values from DFT calculations of pure B-COF, B-COF + 2Li^+^ (with excess Li^+^), and COF-303 + Li^+^ (charge-neutral COFs with one excess Li^+^).**

|  | **B-COF** | **B-COF + 2Li^+^** | **COF-303 + Li^+^** |
| --- | --- | --- | --- |
| X | -2880.29 | -2895.30 | -2906.63 |
| Li | -7.28 | -7.28 | -7.28 |
| X-Li | -2895.30 | -2902.66 | -2913.99 |
| Binding energy (Hartree) | -0.222 | -0.075 | -0.084 |
| Binding energy (eV) | -6.047 | -2.045 | -2.275 |

To further probe the charge environment driving this transport, we analyzed the electrostatic potential (ESP) distribution on the van der Waals surface of the B-COF framework using Multiwfn software based on DFT-optimized structures. The ESP map (Fig. S28; Table S10) reveals pronounced negative potential regions around the B centers, with minimum ESP values reaching –120 kcal mol^–1^, thereby creating strong attractive sites for Li⁺ ions. The negative charge distribution, arising from the anionic borate linkages, provides electrostatic stabilization and guides Li⁺ migration along the interconnected 3D channels by lowering the desolvation energy and promoting site-to-site hopping. In Li^+^@B-COF, the incorporation of Li^+^ attenuates the ESP gradient, reducing the average binding strength, which facilitates faster diffusion, consistent with observed enhancements in conductivity. This aligns with literature on borate-linked COFs, where such charge distributions enhance single-ion conduction by immobilizing anions while selectively accelerating cation mobility. For comparison, neutral COFs exhibit more uniform ESP (around –20 to +20 kcal mol^–1^), leading to higher barriers and lower transference numbers.


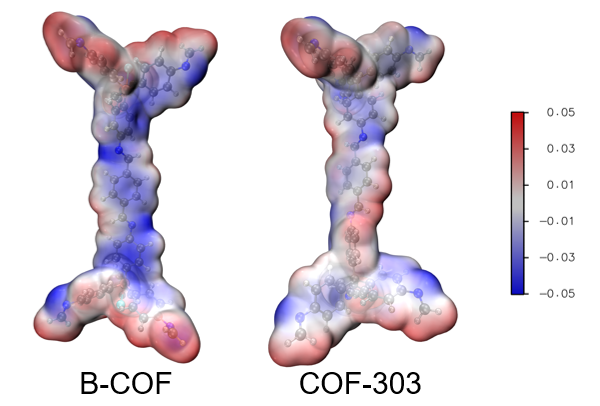


**Figure S28. Electrostatic Potential Distribution (ESP) of B-COF-Li and COF-303.**

**Table S10. Summarized property values of B-COF-Li and COF-303 from the electrostatic potential distribution.**

|  | B-COF-Li | COF-303 |
| --- | --- | --- |
| Global Min. ESP | -1.70 eV | -1.42 eV |
| Negative Avg. ESP | -0.50 eV | -0.43 eV |
| Molecular Polarity Index (MPI) | 0.514 eV | 0.415 eV |
| Polar Surface Area | 494.88 Å^2^ (49.68%) | 411.44 Å^2^ (41.95%) |

**References**

1. Du, Y. *et al.* Ionic Covalent Organic Frameworks with Spiroborate Linkage. *Angewandte Chemie - International Edition* **55**, 1737–1741 (2016).

2. Guo, Z. *et al.* Fast Ion Transport Pathway Provided by Polyethylene Glycol Confined in Covalent Organic Frameworks. *J Am Chem Soc* **141**, 1923–1927 (2019).

3. Jeong, K. *et al.* Solvent-Free, Single Lithium-Ion Conducting Covalent Organic Frameworks. *J Am Chem Soc* **141**, 5880–5885 (2019).

4. Hu, Y. *et al.* Crystalline Lithium Imidazolate Covalent Organic Frameworks with High Li-Ion Conductivity. *J Am Chem Soc* **141**, 7518–7525 (2019).

5. Qiao, Y. *et al.* Application and Research Progress of Covalent Organic Frameworks for Solid-State Electrolytes in Lithium Metal Batteries. *Materials 2023, Vol. 16, Page 2240* **16**, 2240 (2023).

6. Li, X. *et al.* Solution-Processable Covalent Organic Framework Electrolytes for All-Solid-State Li–Organic Batteries. *ACS Energy Lett* **5**, 3498–3506 (2020).

7. Li, X. *et al.* Electrolyte Interphase Built from Anionic Covalent Organic Frameworks for Lithium Dendrite Suppression. *Adv Funct Mater* **31**, (2021).

8. Chen, H. *et al.* Cationic Covalent Organic Framework Nanosheets for Fast Li-Ion Conduction. *J Am Chem Soc* **140**, 896–899 (2018).

9. Li, Z. *et al.* Defective 2D Covalent Organic Frameworks for Postfunctionalization. *Adv Funct Mater* **30**, 1909267 (2020).
